# Overcoming barriers with non-covalent interactions: supramolecular recognition of adamantyl cucurbit[*n*]uril assemblies for medical applications

**DOI:** 10.1039/d3md00596h

**Published:** 2023-12-06

**Authors:** Marija Alešković, Marina Šekutor

**Affiliations:** a Department of Organic Chemistry and Biochemistry, Ruđer Bošković Institute Bijenička 54 10 000 Zagreb Croatia Marija.Aleskovic@irb.hr msekutor@irb.hr

## Abstract

Adamantane, a staple in medicinal chemistry, recently became a cornerstone of a supramolecular host–guest drug delivery system, ADA/CB[*n*]. Owing to a good fit between the adamantane cage and the host cavity of the cucurbit[*n*]uril macrocycle, formed strong inclusion complexes find applications in drug delivery and controlled drug release. Note that the cucurbit[*n*]uril host is not solely a delivery vehicle of the ADA/CB[*n*] system but rather influences the bioactivity and bioavailability of drug molecules and can tune drug properties. Namely, as host–guest interactions are capable of changing the intrinsic properties of the guest molecule, inclusion complexes can become more soluble, bioavailable and more resistant to metabolic conditions compared to individual non-complexed molecules. Such synergistic effects have implications for practical bioapplicability of this complex system and provide a new viewpoint to therapy, beyond the traditional single drug molecule approach. By achieving a balance between guest encapsulation and release, the ADA/CB[*n*] system has also found use beyond just drug delivery, in fields like bioanalytics, sensing assays, bioimaging, *etc.* Thus, chemosensing in physiological conditions, indicator displacement assays, *in vivo* diagnostics and hybrid nanostructures are just some recent examples of the ADA/CB[*n*] applicability, be it for displacements purposes or as cargo vehicles.

## Abstract 2

Supramolecular host–guest systems consisting of adamantanes and cucurbit[*n*]urils have found medicinal applications in drug delivery, controlled drug release, bioanalytics, sensing assays, bioimaging, diagnostics, hybrid nanostructures and beyond.

## Graphical abstract

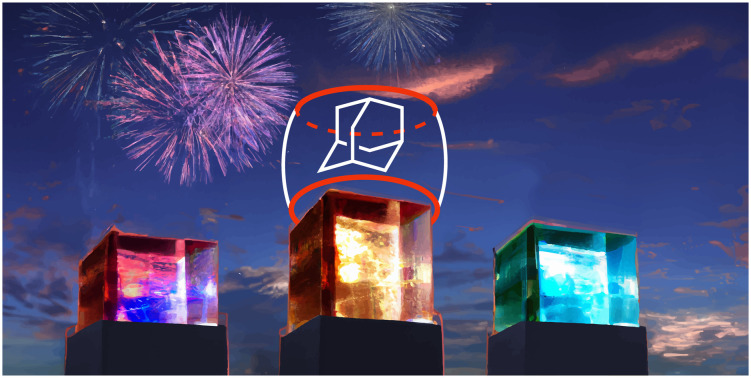

## 1. Introduction to the adamantane scaffold

Adamantane is a fascinating molecule. It has attracted the interest of chemists since it was first discovered and isolated from crude oil in 1933.^1^ After its first synthesis by Vladimir Prelog and Rativoj Seiwerth in 1941 (ref. 2 and 3) and later synthetic scale-up utilizing the Lewis-acid catalyzed rearrangement protocol perfected by Paul von Ragué Schleyer,^4,5^ the chemistry of adamantane^6,7^ virtually exploded and the molecule became available on an industrial scale. Potential of the adamantane cage as a scaffold in medicinal chemistry was realized soon after and in parallel to synthetic endeavors research on adamantane derivatives also took a pharmacological direction.^8–11^ Consequently, the question emerges why adamantane is such a good candidate for bioactive compounds. The answer lies in its structure and the consequences it has on the compound's physicochemical nature. Adamantane (C_10_H_16_) is the smallest homologue of diamondoids,^12,13^ naturally occurring^14^ cage hydrocarbons that possess the structural arrangement of their carbon atoms comparable to a diamond crystal lattice. Adamantane is thus characterized by its high (*T*_d_) symmetry and many exciting properties, like low molecular strain, high thermodynamic stability, high lipophilicity, *etc.*,^7^ combined with vast possibilities of cage functionalization with both donor and acceptor functional groups.^6,7,15^

Application of the adamantane scaffold in medicinal chemistry has a long history dating back to the 1960s when 1-aminoadamantane was discovered to be active against influenza A,^16^ however, to detail every aspect of its utility is outside the scope of this review. A general feature important to note is that the adamantane cage structure is commonly used as a bulky lipophilic addition to a complex structure of an already bioactive molecule since it enables easier crossings of drugs across cell membranes and can also enhance their positive metabolic profiles.^9,11^ However, adamantane's use as a valuable subunit for enhancing properties of known pharmacophores is not its only strength; some simple adamantane derivatives can also have medicinal applications. For example, amantadine (1-aminoadamantane), rimantadine (1-(1-aminoethyl)adamantane) and memantine (1-amino-3,5-dimethyladamantane), simple amine derivatives, found use against viral infections (influenza A, herpes simplex) and neurodegenerative disorders (Parkinson's and Alzheimer's disease).^11^ The antiviral mode of action of simple adamantane amines consists of ion channel blocking of the viral transmembrane proteins,^17^ effectively preventing further virus replication,^18^ while the application as neuroactive compounds is based on a high lipophilicity of adamantane that enables efficient crossing of the molecules across the blood–brain barrier.^19^ Over the years in our laboratory we also explored many adamantane-based compounds as potential bioactive molecules, ranging from applications in enzyme inhibition^20,21^ and antitrypanosomal activity^22^ to their liposome incorporation as a means for targeted compound delivery.^23–25^

In recent times adamantane derivatives have started to attract focus as components of complex drug delivery systems and this exciting new research direction is the main topic of this review. However, before we turn our attention to practical applications, we first need to mention a few key features of the adamantane scaffold that are important in the context of host–guest chemistry. Namely, the adamantane cage is long known to be an efficient scaffold in supramolecular recognition processes, both in chemical and biological systems.^26^ Going from our own experience, adamantane and similar cage compounds have demonstrated an amazing versatility and can serve as a backbone in various cation^27–34^ and anion receptors^35–44^ as well as being desirable guest molecules in inclusion complexes.^45–51^ While in macrocyclic receptors the adamantane cage usually serves as a rigid backbone that reduces the conformational mobility of a macrocycle and thus pre-organizes the receptor molecule for binding, in the inclusion complexes the role of adamantane is primarily limited to enabling preferential binding of the polycyclic subunit into a host molecules. Exactly this host–guest behavior of adamantane derivatives will be the focus of our continued discussion, as it provides a useful tool for recognition in biological systems.

It is known that adamantane derivatives excellently bind to a β-cyclodextrin host and afford stable 1 : 1 inclusion complexes, with association constants typically between 10^3^–10^5^ M^−1^.^24,50,52–57^ Cyclodextrins (CDs) are macrocycles consisting of glucose monomers and are widely used in drug delivery since they are water-soluble, naturally occurring, cheap, and able to bind a wide range of substrates.^58^ Important to note is that cyclodextrins have a well-defined central hydrophobic cavity that is crucial for successful adamantane incorporation and formation of a strong inclusion complex. A good fit between the β-cyclodextrin cavity and the lipophilic adamantane cage thus enables optimal engagement in non-covalent interactions between the two molecules. This behavior has important implications for potential medicinal use since complexation can facilitate better drug molecule solubility and focused delivery to a biological target.^59^ However, widespread use of cyclodextrin for this purpose is still somewhat hampered because of difficulties in membrane crossings of the resulting complexes, often too low binding constants for quantitative drug delivery and too pronounced hydrophilic nature of the host mantle itself. On the other hand, in recent years a different host molecule class, cucurbit[*n*]urils, appeared in the scientific spotlight, also capable of efficient non-covalent binding with adamantane derivatives.

## 2. Properties and molecular binding of cucurbit[*n*]urils

**Fig. 1:**
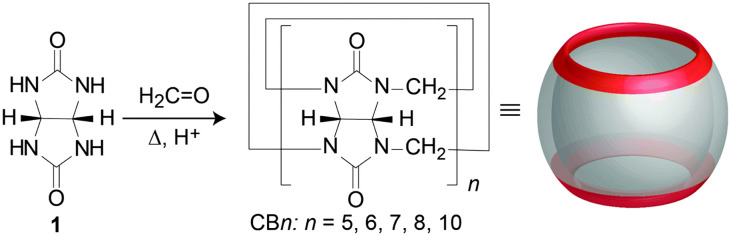
Synthesis of CB[*n*]s from glycoluril and formaldehyde under acidic conditions. Reproduced with permission from ref. 63, the Royal Society of Chemistry, 2015.

In the beginning of the 20th century Behrend *et al.* synthesized a very stable compound from glycoluril and formaldehyde in acidic reaction conditions.^60^ Based only on elemental analysis the authors suggested that this amorphous insoluble polymeric material consisted of three glycoluril molecules condensed together with twice as much equivalents of formaldehyde. However, the correct structure of the obtained molecule remained unknown up until 1981 when Freeman and Mock,^61^ intrigued by this exceptionally thermodynamically stable compound, revised its synthesis. Using modern spectroscopic techniques like NMR and X-ray analysis they elucidated that the stable material in question actually consisted of six glycoluril building blocks interlinked by twelve methylene bridges. The authors also proposed the trivial name “cucurbituril” for the compound because of its structural resemblance to a pumpkin (*Cucurbitaceae* family), as well as to a cucurbit, a gourd-shaped flask that was a common component of the alchemic distillation equipment.^61^ Using modern terminology, we nowadays call this compound cucurbit[6]uril or CB[6] or sometimes designated as CB6, Q[6], Q6 or Cuc6, where the number in the brackets represents the number of glycoluril subunits of the macrocycle (*i.e.*, here a hexamer). Cucurbit[6]uril is thus the first described representative of the cucurbituril family of compounds. In early 2000s Kim reported the synthesis of three other macrocycles from the class that differed in their size, namely CB[5], CB[7] and CB[8].^62^ The trick to obtain other CB[*n*]s was in lowering and controlling the temperature of the reaction which afforded these kinetic products (Fig. 1).

It is curious to note that the discovery of both cyclodextrin and cucurbituril macrocycle families have a similar timeline. However, the studies on CDs have remained consistent over the century whereas the field of cucurbituril chemistry blossomed almost a hundred years after their first synthesis, following the discovery of the mentioned new homologues. Possible reasons for such a slow progress of CB[*n*] chemistry could be ambiguous and somewhat inconsistent synthetic and purification procedures that only recently became systematic,^62,64,65^ and that enabled their commercial availability. In addition to the parent CB[*n*] compounds, other derivatives have been developed as well,^66,67^ like long chain substituted compounds,^68^ twisted^69,70^ and inverted scaffolds,^71^ open-cavity variations,^72^*etc.*

**Fig. 2:**
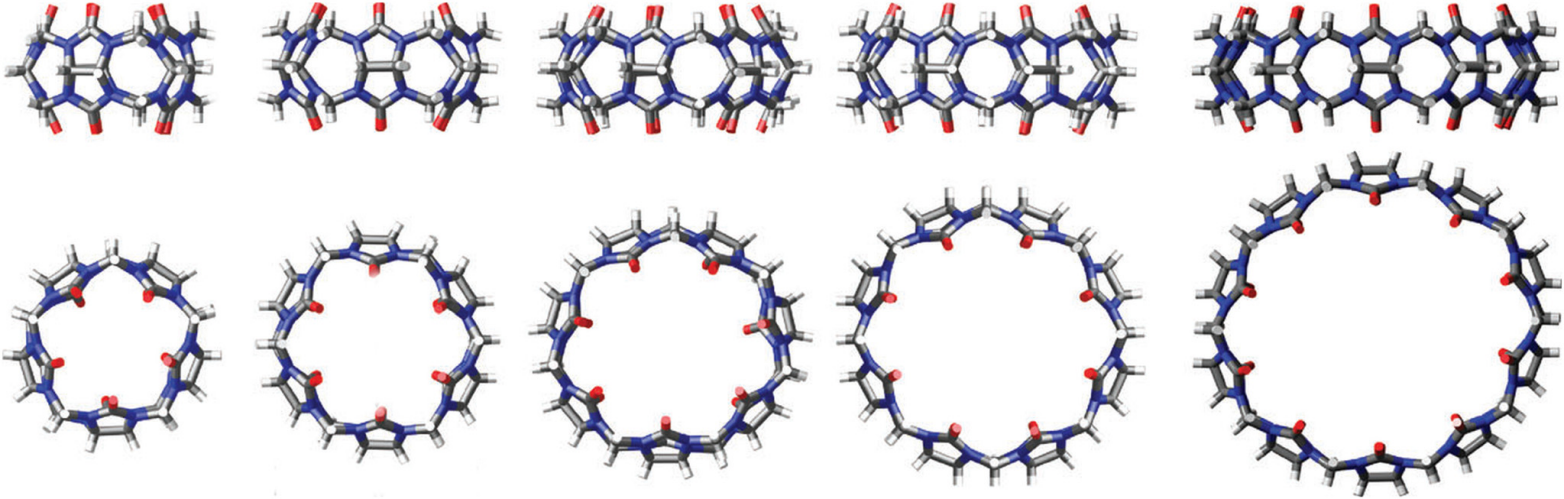
Representations of X-ray crystal structures for CB[*n*] (*n* = 5, 6, 7, 8, 10, left to right). Reproduced with permission from ref. 75, the Royal Society of Chemistry, 2009.

A common feature of all CB[*n*] and similar host scaffolds mentioned earlier is the existence of a deep, centrally positioned cavity. This structural attribute is essential for the accommodation of guest molecules and the formation of inclusion complexes. Ability of CB[*n*]s to complex small organic molecules was noted soon after their rediscovery in 1980s^73^ and from that point onwards the supramolecular chemistry of CB[*n*]s was developing at an astounding rate and scale.^63,74–80^ Despite the most common stoichiometry of the complexes between CB[*n*]s and the guest molecules being 1 : 1, CB[10]^81,82^ can accommodate even two guest molecules within its cavity. Curiously, CB[10] itself was first isolated as a CB[5]@CB[10] complex.^81^ The complex strength, *i.e.*, effectiveness of the binding, is expressed through values of the stability constants which are basically the ratio of the rate constant for the complex formation (ingression) and dissociation (egression). It should also be mentioned that CB[*n*] homologues CB[5]–CB[10]^61,62,75,81^ have all been fully characterized by X-ray crystallography (Fig. 2), providing key structure parameters that are summarized in Table 1.

**Table 1:** Structural parameters (CB[5],^62^ CB[6],^61^ CB[7],^62^ CB[8],^62^ CB[10]^75,81^) and physicochemical properties for cucurbit[*n*]urils

CB[*n*]	CB[5]	CB[6]	CB[7]	CB[8]	CB[10]
Outer diameter/Å	13.1	14.4	16.0	17.5	20.0
Inner cavity diameter/Å	4.4	5.8	7.3	8.8	11.7
Portal diameter/Å	2.4	3.9	5.4	6.9	10.0
Inner cavity volume/Å^3^	84	162	279	479	691
Height	9.1	9.1	9.1	9.1	9.1
Number of water molecules in cavity^84^	2	4	8	12	22
Solubility in water^74,75^/mM	20–30	0.02	20–30	<0.01	<0.05 (ref. 82)
Stability^74^/°C	>420	425 (ref. 85)	370	>420	

All the mentioned unmodified CB[*n*]s have the height of 9.1 Å, which is similar to the depth of the CDs, whereas by adding more glycoluril units to the macrocycle ring the cavity diameter and its volume increase (Fig. 2). As can be seen from Fig. 2, on the top and the bottom of the cavity two hydrophilic carbonylated rims are symmetrically positioned. The portals are approximately 2 Å narrower than the cavity, enabling discrimination of the guest molecules based on their size and thus accomplishing selectivity. Additionally, such doubly pinched geometry provides a barrier to association and dissociation of guest molecules. Because of the electronegative nature of the portals, cationic guests form with CB[*n*] hosts inclusion complexes that are in principle of higher affinity than is the case for comparably sized neutral guest molecules.

As it was noted right from their first synthesis,^60^ CB[*n*]s are unusually thermally stable molecules, while their main drawback is their somewhat poor water solubility. Curiously, CB[*n*] macrocycles with an odd number of glycoluril subunits (*n* = 5, 7) are more soluble in pure water than CB[*n*]s with an even number (*n* = 6, 8, 10). This can be explained by stabilization of the crystal lattice with multiple close and strong contacts between macrocycles which outnumber the close contacts of CB[*n*] with water. Water solubility can be enhanced by adding acids or ionic salts to the medium or upon encapsulation with amphiphilic guests. Quite recently it was found that their limited solubility can also be overcome by mechanochemical synthesis of host–guest complexes with CB[7].^83^

**Fig. 3:**
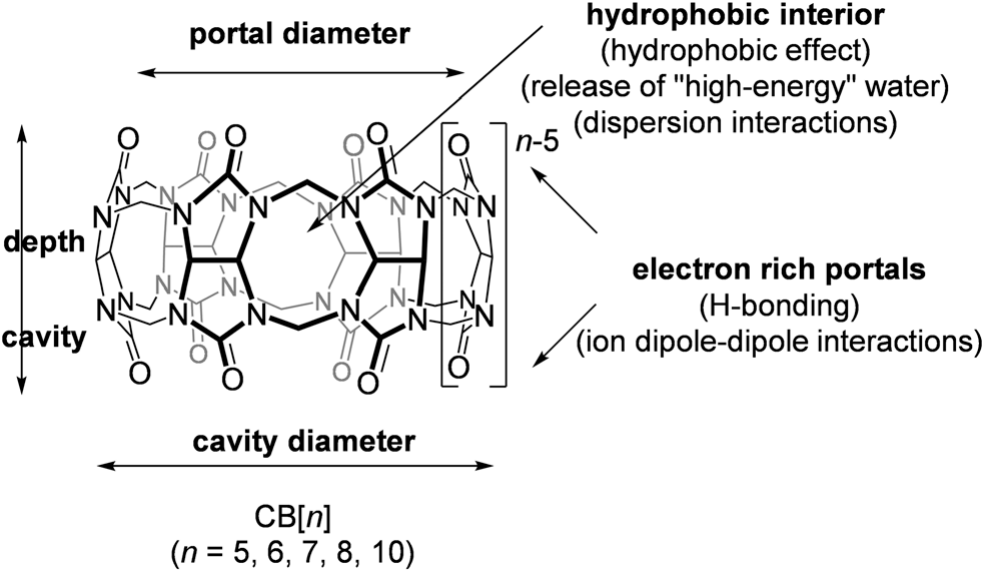
CB[*n*]s regions important for molecular recognition.

Every region of these exceptional macromolecules has its function in molecular recognition processes and enables a good overall fit and ultimately the formation of a thermodynamically stable complex (Fig. 3). Beside intrinsic characteristics of the host and guest molecules, the supramolecular system also depends on the surroundings, making the binding event and complex stability sensitive to a change of solvent, temperature, ion strength, pH, *etc.*^86–88^ Moreover, these supramolecular interactions and macrocyclic characteristics can lead to additional phenomena such as change in intrinsic properties of the guest molecule (*e.g.*, higher solubility), improved chemical and thermal stability, *etc.* For example, it was found that when a protonated basic guest forms inclusion complexes within the CB[*n*] cavity the p*K*_a_ value of the guest becomes greater, demonstrating a different preference for binding of a protonated form when compared to the neutral one.^89^ Complexation induced shifting of the p*K*_a_ values of the guest molecules has already found applications in sensors,^90–94^ assays,^95,96^ as well as in drug delivery systems.^97–99^

**Fig. 4:**
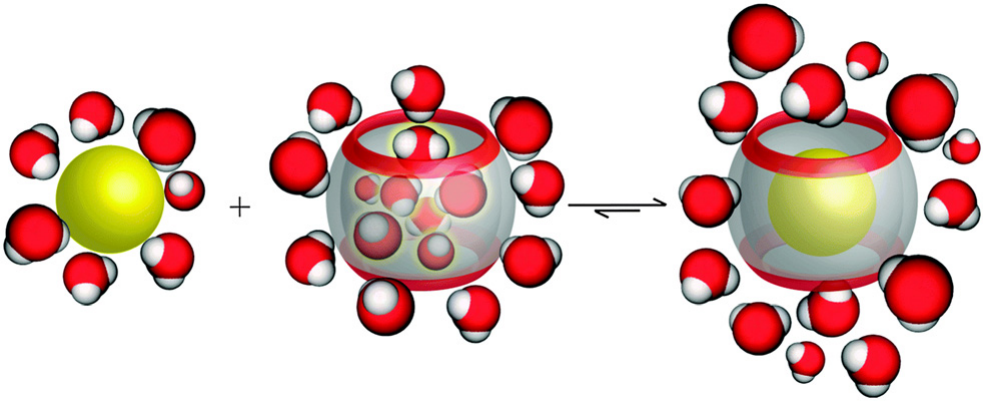
Depiction of the release of high-energy water molecules from the CB[*n*] cavity upon binding of a hydrophobic guest. Reproduced with permission from ref. 63, the Royal Society of Chemistry, 2015.

On another note, it was postulated that the optimum occupancy of the CB[*n*] host interior for the inclusion of guest molecules would be 55 ± 9%.^100^ In that case physicochemical properties of the guest molecule such as size and its shape to appropriately fit into the host interior could be predictors of selectivity and binding intensity.^84^ However, there have been examples where packing coefficient is greater or smaller than supposedly optimal and in those cases additional interactions emerge to compensate for the postulated non-ideal packing inside the cavity.^45,101^ Here should also be noted that the cavity of the “empty” host is not actually empty in any real world system, since it readily interacts with solvent molecules. In fact, CB[*n*]'s interior can host between 2 and 22 water molecules, depending on its size. In the case of aqueous solutions, water molecules occupying the cavity of low polarity are somewhat isolated from bulk water molecules and as a consequence cannot be optimally stabilized through hydrogen bonds as would be the case in the bulk water environment.^102^ The release of these so-called “high-energy” water molecules upon encapsulation of the guest molecule is actually one of the primary driving forces for complex formation and was proven to be responsible for strong affinities (Fig. 4).^84,103,104^

Interactions such as the hydrophobic effect and dipole–dipole or ion–dipole interactions between the guest molecules and the electronegative CB[*n*] portal rim are other important stabilizing factors during the binding event. Dispersion interactions are also to be considered for binding because they can provide an additional boost in the overall association constant values despite their weaker individual strength when compared to other interaction types. All these interactions and effects are synergistic and can result in exceptionally stable complexes with high association constants that were previously rarely or never observed. Such extremely stable complexes can even rival the avidin–biotin affinity,^105,106^ which is known to be one of the strongest non-covalent interactions between two species found in nature.^45,107–111^ In continuation we will see how these exceptional characteristics of CB[*n*]s can be utilized for pharmacologically applicable supramolecular host–guest assemblies.

## 3. Cucurbit[*n*]urils in drug delivery

Supramolecular chemistry provides a versatile platform for creating complex structures with applications ranging from drug delivery, therapeutic interventions to diagnostics. Molecular recognition and encapsulation of hydrophobic species in a macrocycle's cavity (especially in aqueous environment) is perhaps the most important advantage of cyclic macromolecules like cyclodextrins,^112^ pillar[*n*]arenes,^113^ cucurbit[*n*]urils, calixarenes,^114^*etc.* Such cyclic host molecules are therefore an excellent alternative to other popular drug delivery systems like micelles,^115,116^ dendrimers,^117^ liposomes,^118^*etc.*, mainly because of the former's thermal and chemical stability, availability of different cavity sizes and the ability to self-assemble into various nanostructures. Macrocyclic carriers in general have well defined binding ability both *in vitro* and *in vivo* and the bioactive guest molecule encapsulated in the cavity is protected against inactivation or metabolic degradation, leading eventually to a controlled drug release. In other words, the macrocyclic cavity can be viewed as the binding region of a biological receptor which has a pre-organized shape for the formation of a host–guest complex. This biomimetic function, along with their biocompatibility, intrigued medicinal chemists for a long time and Freudenberg *et al.* first patented the idea of using cyclodextrins for the inclusion of physiologically active organic compounds as early as the 1950s.^119^ However, it took almost 25 years after that initial idea until the first cyclodextrin-containing pharmaceutical product was actually marketed.^120^ Nevertheless, from that point onwards cyclodextrin drug carriers have been constantly developing and currently there are about 35 different approved CD-containing drug products available,^120–122^ with the overall number of drug containing macrocycles continuously growing.^123,124^ In fact, usage of a known pharmaceutically active compound which upon binding^125^ demonstrates enhancement of its initial properties in terms of stability, solubility, *etc.* is often less time consuming and has economic advantages over the search and testing of new potential candidates. Modulation of chemical and physical parameters of bioactive species in inclusion complexes will be discussed later in more detail.^98,126^

As for the cucurbit[*n*]urils, after optimization of their synthesis,^62^ isolation^62,64^ and purity analytics,^127^ studies on this macrocycle family have initially been focused mostly on their molecular recognition properties.^73^ However, that shifted in recent times towards potential application, especially as even more economical synthesis procedures have been developed, *e.g.*, by using microwave techniques^128,129^ or through solvent free mechanochemistry methods.^130^ Nowadays CB[*n*]s have taken over the prominent spot of highly promising macrocyclic candidates for biological applications and drug delivery.^98,122,125,131–151^ Moreover, CB[*n*] hosts have shown potential as supramolecular antidotes, neutralizing the effects of a poison though the encapsulation of a small toxic molecule.^147,152,153^ The so-called chemical reasons for these new trends are the observations that CDs, when compared to CB[*n*]s, show generally lower selectivity and weaker binding affinities^52^ (*K*_a_ are usually lower than 10^5^ M^−1^). It follows that in order to achieve quantitative binding of the guests, one needs to use much larger concentrations of CDs, while CB[*n*]s possess inherently stronger potential for efficient binding. In other words, because of the high stability constants only small amounts of free uncomplexed CB[*n*] molecules will be present in solutions, with >99% of drug complexation being achieved. Note however that not all homologues of the CB[*n*] series are equally suitable as drug carriers; CB[5] is too small to encapsulate drug molecules, while CB[10] is often too large to produce high affinity complexes. CB[6], CB[7] and CB[8] are therefore at present the most commonly considered analogues for bioapplications and in drug delivery systems.

As already mentioned in the introduction, many adamantane (ADA) derivatives have been used as drugs^10^ for decades and the realization that the adamantyl cage almost ideally occupies the corresponding cucurbituril cavity instantly led to studies on how this host–guest supramolecular system could affect bioactivity and bioavailability of the drug molecule as well as tune drug properties. Moreover, it was also explored how the ADA/CB[*n*] supramolecular interaction could be implemented in fields other than just drug carrier design, like in sensing, general biology and medicine in a broader sense. We will therefore present in this targeted review illustrative examples of ADA/CB[*n*] use in biological applications, with special focus not only on the straightforward drug-in-a-carrier cases but also on broader examples of bioactive supramolecular systems benefiting from this extraordinary supramolecular interaction.

Using hydrophobic ADA in combination with CB[*n*]s is a sort of a golden standard in this field because the scaffold in question fits so snugly into the central cavity of the host,^108,109^ thus accomplishing strong and selective binding. The consequence is that the ADA/CB[*n*] system is much more efficient at achieving total encapsulation than is the case for many other guest molecules.^154^ The binding preferences of various ADA derivatives for CB[*n*]s have been explored thermodynamically, structurally and electronically,^109^ thereby making most of the aspects of its molecular recognition in supramolecular processes known. Table 2 presents proof-of-concept association values for complex formation with two commonly used ADA derivatives in a 50 mM NaO_2_CCD_3_-buffered D_2_O (pD 4.74), as determined by ^1^H NMR competition experiments.

**Table 2:** Values of *K*_a_ (M^−1^) for the interaction of amantadine and memantine with CB[7] and CB[8]^109^

Compound	CB[7]	CB[8]
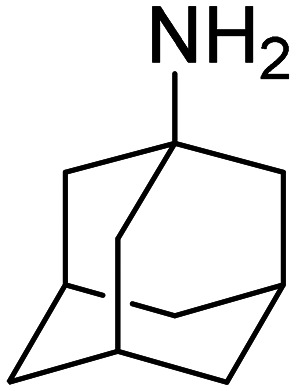 Amantadine	(4.23 ± 1.00) × 10^12^	(8.19 ± 1.75) × 10^8^
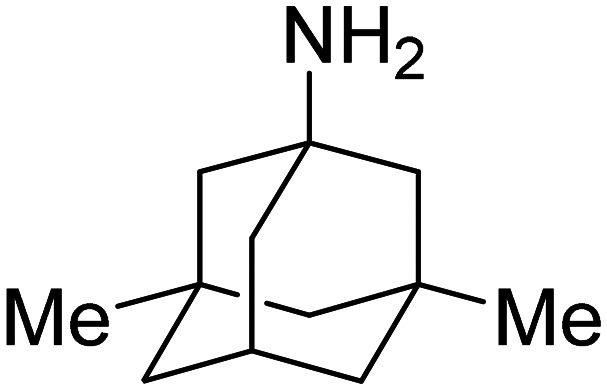 Memantine	(2.50 ± 0.39) × 10^4^	(4.33 ± 1.11) × 10^11^

When comparing amantadine (1-aminoadamantane) affinities towards CB[7] and CB[8], it can be seen that the binding preference is significantly in favor of CB[7] (Table 2). This is presumably because of the optimal structural packaging and better complementarity of the ADA scaffold and the CB[7] cavity, resulting in much higher *K*_a_ value. At the same time, the opposite is observed for the binding of a trisubstituted ADA derivative memantine (1-amino-3,5-dimethyl-adamantane), *i.e.*, memantine/CB[8] is 10^7^-fold tighter than the complex of memantine/CB[7]. The most probable explanation is that two additional methyl substituents on the ADA cage destabilize the complex that forms with a smaller CB[7] due to unfavorable steric interactions between the convex surface of the guest and the concave cavity wall surface of the host. Nevertheless, CB[*n*]-type macrocycles retain a relatively high binding affinity towards both amantadine^155^ and memantine.^156^

The charge present on protonated adamantane derivatives introduces electrostatic interactions with the CB[*n*]'s rim, enhancing the binding affinity to CB[*n*] compared to neutral adamantane compounds, which inspired Isaacs and coworkers to test how the observed trend would affect mechanical features of the two complex types.^48^ They constructed an assembled DNA template in which a flexible DNA linker was exploited to keep the CB[7] and ADA in close proximity. It was found that positively charged ADA guest possesses higher mechanical stability (49 pN) than neutral adamantane (44 pN) holding on to CB[7], which falls in the strength range of many biological recognitions. Furthermore, it was found that a hexyl group adjacent to the ADA moiety served as a chaperone to assist the formation of the ADA–CB[7] pairs, which was not observed with ADA/CD complexes.^157^

**Fig. 5:**
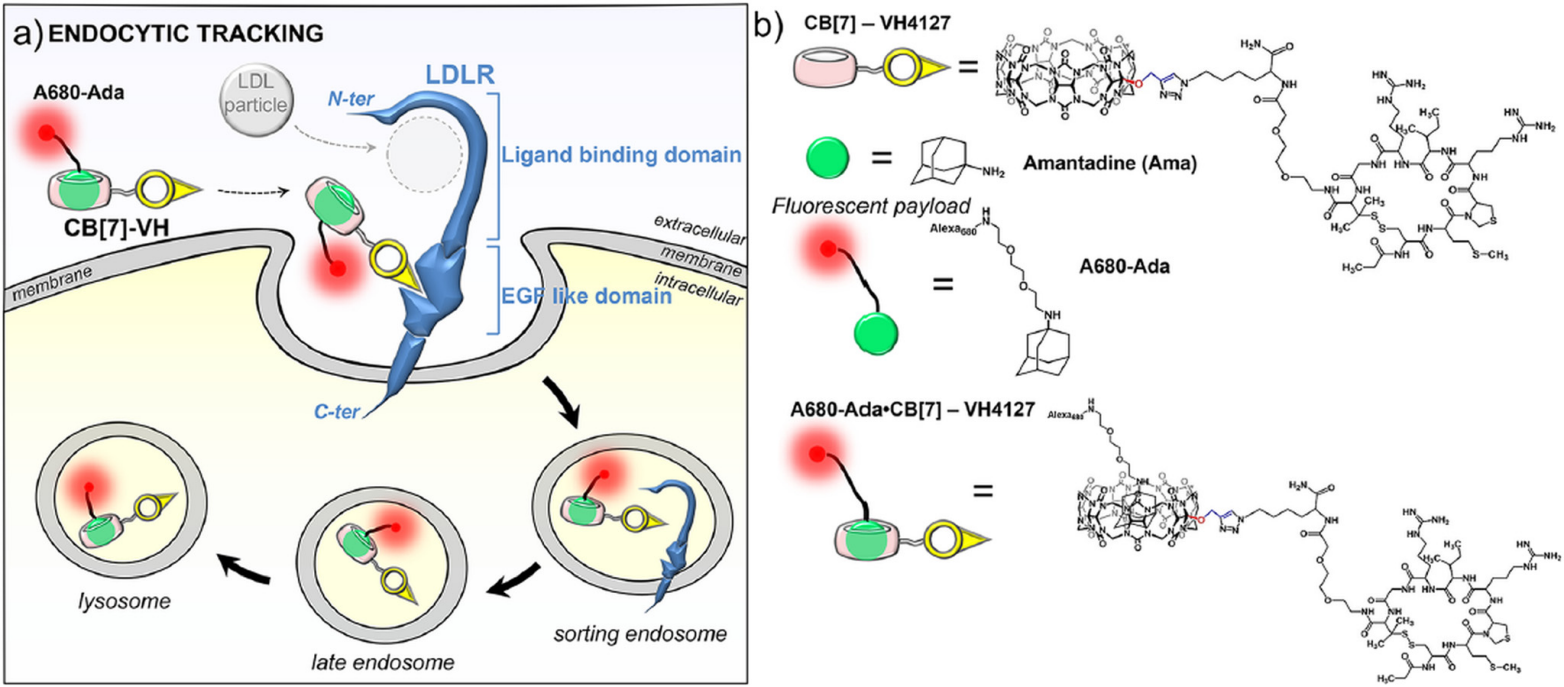
Illustration of non-competitive binding of a supramolecular complex consisting of an ADA-derivative and peptide-functionalized CB[7] to a low density lipoprotein receptor (LDLR). Reproduced with permission from ref. 158, the American Chemical Society, 2023.

Extraordinarily stable complexes formed between the adamantyl group and the CB[*n*]-type macrocycles enable implementation of this supramolecular system in tracking pathways and intracellular delivery since almost quantitative complexation occurs at sub-nanomolar concentrations. An excellent example that illustrates this point very well was reported very recently, dealing with evaluation of the uptake potential of cyclic peptide (VH4127) connected to CB[7] that non-competitively targets the low density lipoprotein (LDL) receptor (LDLR) in order to achieve a new kind of drug delivery system (DDS) (Fig. 5).^158^ In this context an analogous conjugate was synthesized which comprised of ADA coupled with a fluorescent group and the resulting supramolecular complex retained binding to LDLR and even improved LDLR-mediated endocytosis and intracellular accumulation potential in LDLR-expressing cells. The authors concluded that such an approach expands the design possibilities of a wide range of therapeutic or imaging vehicles through utilization of the transport abilities of the CB[*n*] macrocycle.

However, one must carefully design the system in which ADA/CB[*n*] will be incorporated and estimate whether the final assembly would be too bulky and/or hydrophilic to enter the cells in sufficient amount as it was demonstrated in the case of four CB[7]s that were non-covalently attached to aminoadamantyl-substituted phthalocyanines (Pcs).^159^ The study in question showed that supramolecular interaction between CB[7] with Pcs in water improved both fluorescence and singlet oxygen production. On the other hand, bioactivity decreased for at least an order of magnitude after host–guest interaction with CB[7], which was attributed to much lower uptake by cells due to the aforementioned reasons.

### 3.1. Bioactivity, toxicity and membrane crossing

From a bioapplicability perspective, CDs can be nephrotoxic^160^ if they enter the body in a non-metabolized state, thus precipitating as complexes with cholesterol.^161^ On the other hand, CB[*n*]s demonstrated lower toxicity in preliminary assays.^162–166^ In order to assess bioavailability and toxicity of CB[*n*]s, first it has to be determined whether the host–guest complexes can cross cell membranes. Afterwards, it needs to be estimated if CB[*n*] itself is (non)toxic and whether the CB[*n*] complex combined with the drug is (non)toxic as well. Finally, it needs to be determined whether the drug inclusion in CB[*n*] decreases the toxicity side-effects of the drug itself. Ideal function and safety requirements of the envisioned macrocyclic host imply that it would carry the drug molecule to a desired location within the body, crossing barriers and delivering the active compound at a defined concentration, preferably through a slow, sustained release. At the same time it would be desirable that it does not decrease the activity of the drug towards targets in the organism. Finally, the carrier itself has to be efficiently excreted from the body, either unchanged or in a metabolized form. With those desirable traits in mind, hemocompatibility of erythrocytes and leukocytes with CB[*n*]s was investigated and no cytotoxic effect was observed even at high concentrations (1 mM).^167^ Even though CB[*n*]s do not damage cells directly, enhancement of the early apoptosis of lymphocytes was noticed, thus, CB[7] enhances hemolysis in biological media. Despite this finding, the authors concluded that CB[*n*]s are still fairly safe organic molecules to be used in lower concentrations. Immunotoxicity and immunomodulation properties of CB[*n*]s were also tested and it was found that CB[7] and CB[6] did not decrease the viability of mononuclear cells at all tested concentrations (0.1–1 mM range). Furthermore, the results indicated an immunomodulatory effect of different concentrations of CB[*n*] and after a longer cultivation time CB[*n*] had an immunostimulating effect, which was indicated by an enhancement of the proliferative activity of cells and increased expression of HLA-DR on lymphocytes.^168^

**Fig. 6:**
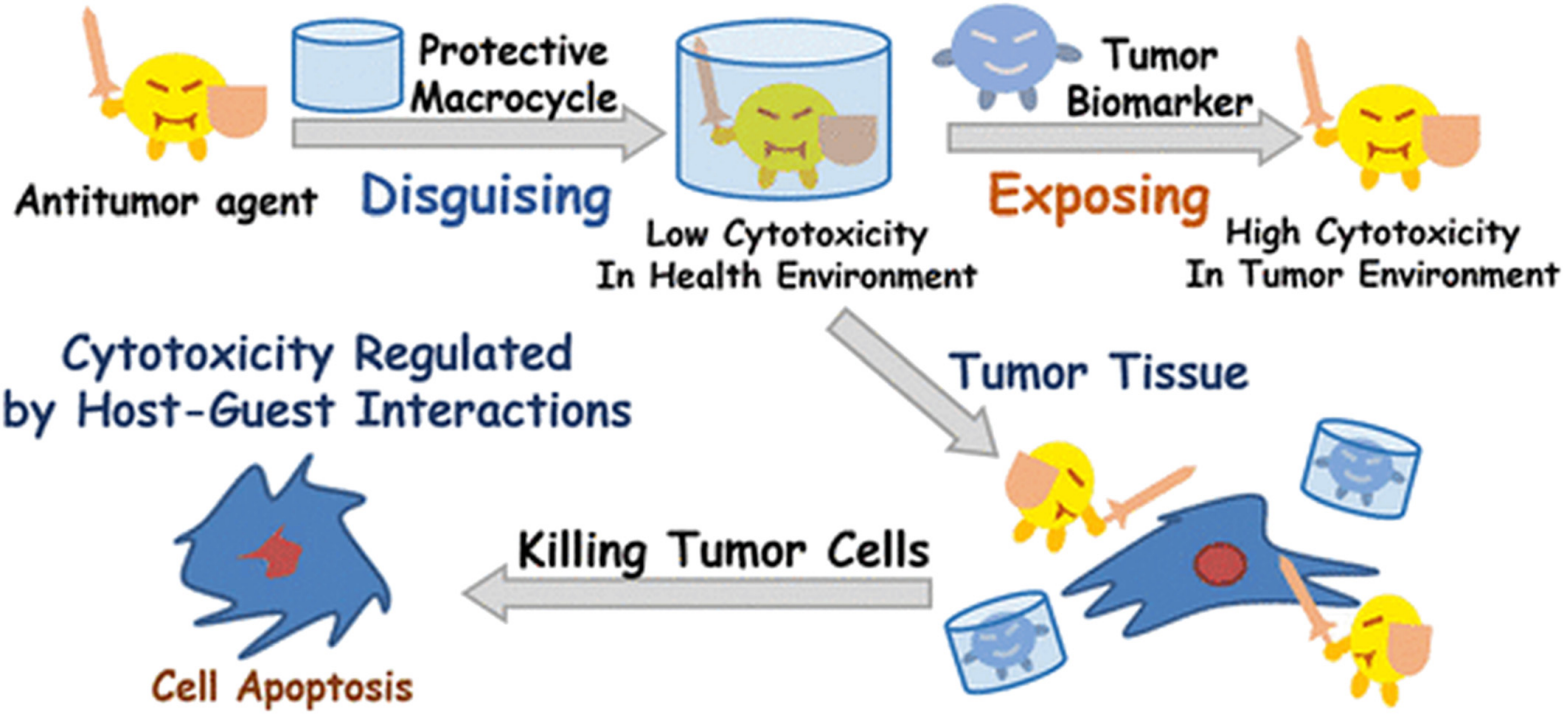
Schematic representation of supramolecular chemotherapy with a goal to decrease cytotoxicity and increase antitumor activity by host–guest chemistry. Reproduced with permission from ref. 169, the American Chemical Society, 2016.

Since CB[7] has been the most studied representative of the CB[*n*] family in terms of potential biological application, biocompatibility and safety of CB[7] was systemically evaluated *in vitro*, *ex vivo*, and *in vivo* using animal and human cell lines, zebrafish and in mice.^162–166^ It was shown in several *in vivo* studies that there is little or no obvious toxicity when the cell lines were treated with CB[7] at concentrations below 500 μM,^162,163^ which is far above the concentrations typically needed for drug delivery. This means that mice can survive intravenous doses up to 250 mg kg^−1^ of body weight,^162^ which indeed makes CB[7] a promising candidate in leading drug delivery exploration. The *ex vivo* detailed studies showed neurotoxicity, myotoxicity, and cardiotoxicity of CB[6] and CB[7] at 1 mM, 300 μM, and 300 μM, respectively.^164^ What is more, toxicity evaluation of a complex consisting of platinum-based anticancer drug cisplatin and CB[7] on these same tissues was also performed. It was found that the free drug cisplatin displayed toxicity on all examined tissue types, but when encapsulated in CB[7], the myo- and cardiotoxic activities were considerably reduced, while no relevant change in toxicity was observed in the neurotoxicity studies.^164^ Encapsulation of cisplatin in the CB[7] cavity therefore results in a decrease in its cytotoxicity to healthy cells, which could solve the issues caused by known low specificity of chemotherapeutics to cancer cells. This principle of conditioning cytotoxicity in chemotherapy by applying supramolecular systems was adopted as a new strategy aptly named supramolecular chemotherapy (Fig. 6),^169–171^ and is an illustrative proof-of-principle of integrating non-covalent interactions into a synergy of drug design, targeted delivery and combined drug therapy.

**Fig. 7:**
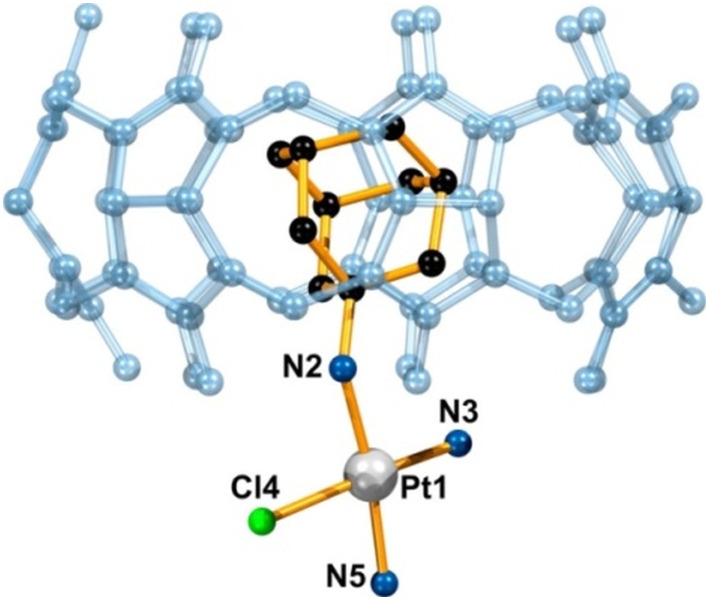
Supramolecular structure of a host–guest complex consisting of a Pt-containing ADA derivative and CB[7] determined by X-ray diffraction analysis. Hydrogens, counterions, and water molecules are omitted for clarity. Reproduced with permission from ref. 173, the American Chemical Society, 2021.

It was previously demonstrated that a Pt(ii) complex with the ADA anchored malonate in a β-CD cavity had higher toxicity toward tumor cells due to its increased DNA binding ability and more efficient cell uptake, which pointed towards advantages of applying supramolecular non-covalent interactions in anticancer therapy.^172^ Quite recently a group of authors^173^ reported a supramolecular host–guest system consisting of cationic platinum(ii) compounds tethered with substituents such as amantadine, rimantadine, and bornylamine and then anchored in the cavity of the CD or CB[*n*] macrocyclic host (Fig. 7). Again, the idea was to improve on the platinum-based anticancer drugs using lipophilic ligands with bulky substituents that are already pharmacologically active by aiming for a more efficient penetration across membranes, reduction of side effects and tumor targeting. Because of the anchoring capability of these Pt-containing compounds and their ability to fit well into the β-CD and CB[7] cavities, the metallodrug guests are protected against rapid degradation. As expected, the authors observed a preference for the formation of more stable complexes for the CB[7] cavitand rather than for β-CD, albeit with a slower exchange between the free and the bound form. What is more, such strong host–guest interaction is responsible for a reduced release of drug molecules from the CB[*n*], resulting in a lower than expected biological response of the system. The authors proposed a solution to this issue by keeping the transport benefits afforded by the host molecule while modifying the release through the lowering of the host affinity towards the guest molecule or through lowering the stability of the linker *via* chemical modification.

**Fig. 8:**
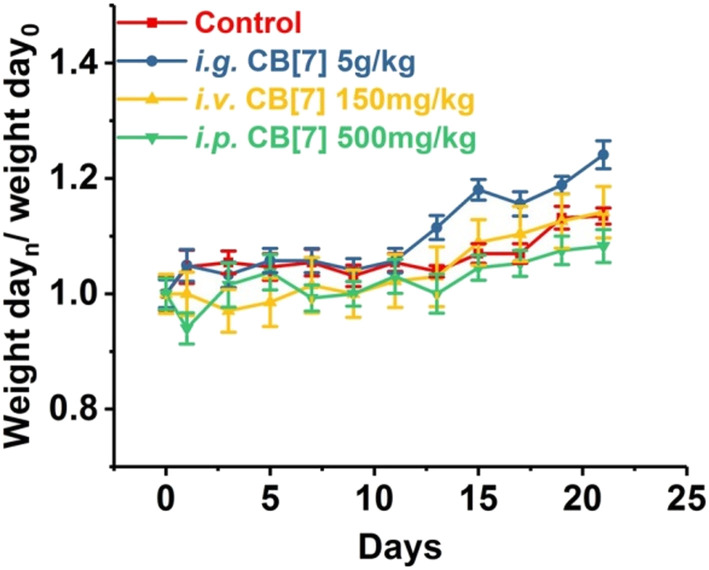
Body weight changes of mice i.g. (orally), i.v. (intravenously) or i.p. (peritoneally) administered with CB[7], respectively, monitored for 21 days post-administration. Reproduced with permission from ref. 166, Nature Research, 2010.

CB[7]'s *in vivo* biocompatibility was also evaluated on a zebrafish model, which revealed a low toxicity of CB[7] to zebrafish.^165^ The results show that CB[7] exhibits measureable cardiotoxicity and toxicity at concentrations of 0.50 mM or higher, without significantly observable change on hepatotoxicity phenotype at concentrations up to 0.75 mM.^165^ Subsequently, a systemic evaluation of the *in vivo* biocompatibility of CB[7] was conducted on a mice model as well.^166^ The maximum tolerable single doses of administered CB[7] on mice were 5 g kg^−1^ orally, 500 mg kg^−1^ peritoneally, and 150 mg kg^−1^ intravenously, respectively. There were no significant differences among various groups of mice in terms of body-weight compared to the control group, although there was a small decrease in body-weight within the first three days (Fig. 8). It was concluded that even at higher doses of administrated CB[7] there were no noticeable toxicity effects.^165,166^

Very recently a systematic evaluation of the CB[7] pharmacokinetics and toxicity was performed to evaluate specific organ toxicity, *i.e.*, on plasma, brain, kidney, and gastrointestinal tract by various methods of injection (intramuscular, intraperitoneal and intragastric).^174^ Only minor liver damage was observed when CB[7] was administered intragastrically. Overall, parenteral administration led to fast elimination from blood and minimal absorption from the gastrointestinal tract and was well tolerated even when repeated. Unfortunately, kidney damage was observed and 3.6% of animals showed signs of nephrotoxicity which may be the most critical drawback of its parenteral use, since most of its elimination from the body is through excretion by kidneys.^174^ Nevertheless, toxicological profiles of CB[*n*]s in general, even acyclic-CB[*n*] and CB[*n*]-type^175^ ones, support the idea of their exploitation in biological applications at subtoxic concentrations. Although CB[*n*]s seem to be promising molecules, long-term side effects from continuous use of CB[*n*]s are still relatively unknown and should be determined, which is also one of the reasons that CB[*n*]s are presently not in clinical use and have not yet been tested in humans.

**Fig. 9:**
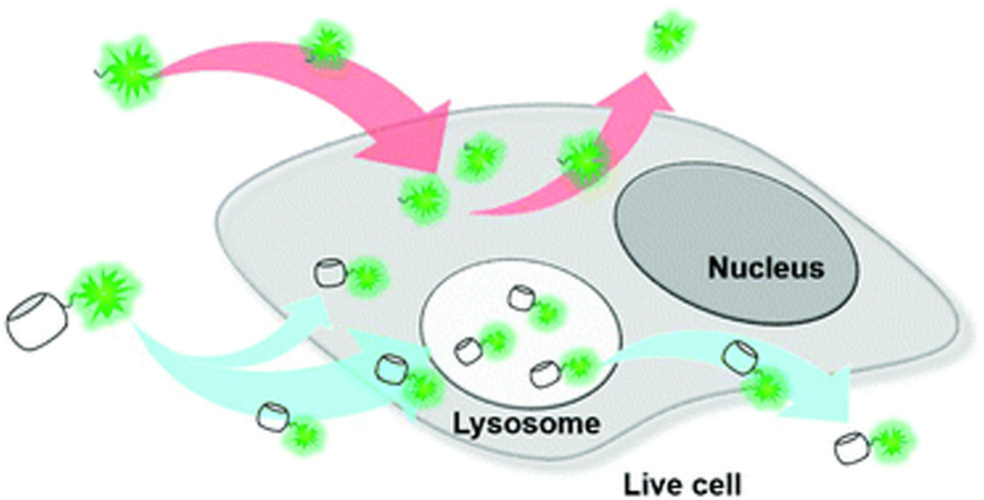
Illustration of intra- and extracellular translocation of dyes. Reproduced with permission from ref. 179, the Royal Society of Chemistry, 2019.

Note that CB[*n*] macrocycles and their complexes successfully cross the cell membranes, which is typically visualized by fluorescence microscopy that additionally confirms their already observed excellent bioavailability. Moreover, even large CB[*n*]-based nanoparticles^176^ (∼190 nm) or CB[*n*]-carbohydrate clusters^177^ can successfully cross the cell membrane by receptor-mediated endocytosis and release the loaded drug into cytoplasm after endocytosis, thereby increasing the pharmaceutical effects of the drugs on a target cell. In another study CB[7] complexes with fluorescents dyes acridine orange and pyronin Y successfully penetrated the cell membrane of mouse embryo muscle cells, demonstrating that other CB[*n*] host–drug complexes are likely to behave *in vivo* in a similar fashion.^178^ CB[7] molecules conjugated to cyanine and rhodamine dyes also displayed uptake into live cells (human breast carcinoma cells, MCF-7) *via* multiple pathways but mainly by clathrin-mediated endocytosis and passive diffusion (Fig. 9).^179^ Most of the CB[7]-dye molecules accumulated in lysosomes were afterwards excreted from cells *via* a lysosome-associated exocytosis, involving the fusion of lysosomes with the plasma membrane rather than Golgi-mediated pathways of excretion for foreign materials.^179^ Note, however, that the free molecules of dyes were excreted by the Golgi mechanism. All these findings demonstrated that the CB[7] moiety indeed had a substantial influence on the uptake and excretion pathways of molecules, making it suitable as a molecular probe for a wide variety of applications in bioimaging.^179^ Even more, by using live-cell imaging it was revealed that treatment with subtoxic CB[7] amounts did not have damaging effects on the cellular integrity, as assessed by mitochondrial activity.^162^

**Fig. 10:**
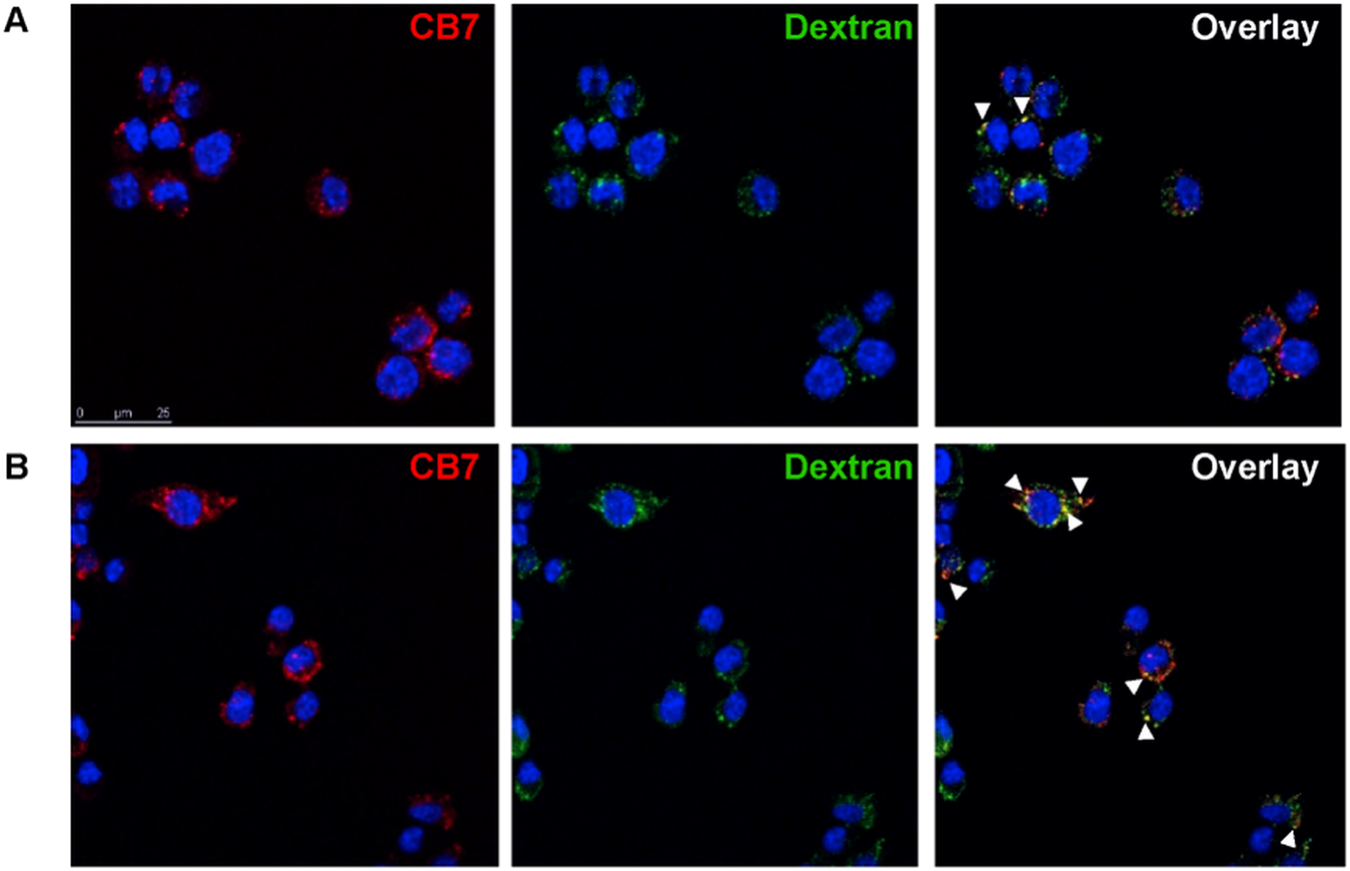
Intracellular localization of CB[7] in RAW264.7 cell. Cell incubated with Dextran-647 and ADA-derivative/CB[7] showed intracellular localization of CB[7] through the endosomal pathway. RAW264.7 cells were incubated with Dextran-647 (green) overnight and ADA-derivative/CB[7] (red) for 20 min the following day. Cells were chased for 15 (A), 45 (B) min after incubation with ADA-derivative/CB[7]. Arrows indicate co-localization. Reproduced with permission from ref. 163, Public Library Science, 2010.

In their toxicity and drug potential study, Isaacs and Briken^163^ synthesized compounds that consisted of a signaling subunit, such as fluorescein or Alexa Fluor 555, that was then attached to spermidine and 1-aminoadamantane subunits in order to facilitate tight binding with CB[*n*] or CB[*n*]-type compounds. Their rationale was that the hydrophobic part of the molecule (ADA or alkyl chain) will provide a good anchor in the CB[*n*] cavity while the fluorescent part of the molecule can be used to track these CB[*n*] molecular containers inside cells in real time. Co-localization assays using fluorescence microscopy showed an uptake of the studied ADA-derivative/CB[7] and Dextran-647 into murine macrophage cells with little co-localization after 15 min (Fig. 10A). However, at 45 min, an increase in CB[7] co-localization with Dextran-647 was observed (Fig. 10B). The complex in question had intracellular stability for more than 2 hours.^163^

**Fig. 11:**
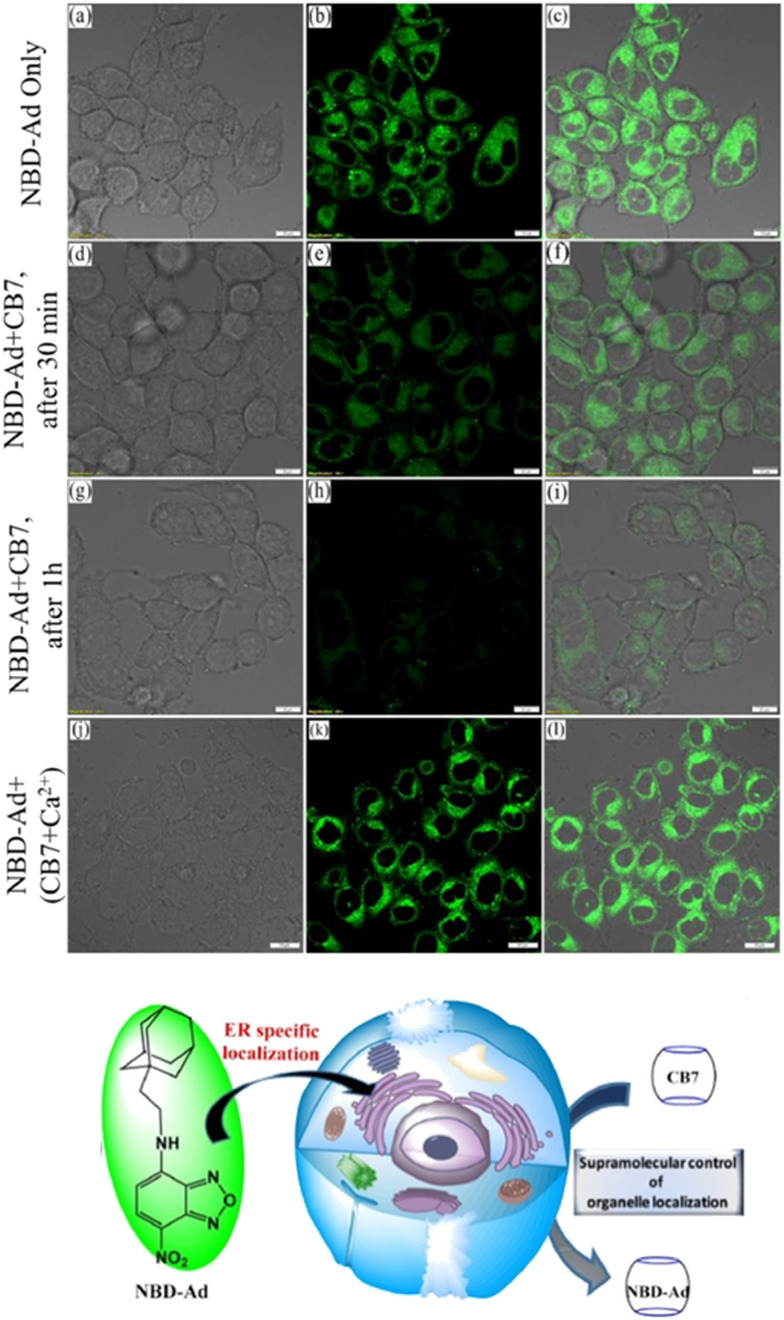
Fluorescence co-localization microscopy images of live HeLa cells stained with 2 μM NBD-Ad (a–c, up), after 30 min of 20 μM CB[7] post-treatment (d–f, middle), after 1 h of CB[7] post-treatment (g–i, bottom), and (j–l) post-treatment with the Ca^2+^/CB[7] complex with a retaining of the NBD-Ad in the ER. Reproduced with permission from ref. 180, Elsevier, 2021.

Recently an ADA derivative coupled with a fluorophore nitrobenzoxadiazole (NBD-Ad) was used as a probe that efficiently labels the endoplasmic reticulum (ER) in living cells (Fig. 11).^180^ CB[7] time-dependent imaging revealed a loss in specificity for ER by observing a reduction in the fluorescence signal, which was a useful proof-of-principle that showed how sub-cellular localization of a probe can be monitored using the supramolecular approach.^180^

### 3.2. Properties of CB[*n*]-drug systems

A comprehensive study of broad chemical, physicochemical and biological parameters should be undertaken before employing a supramolecular system in medicine. Especially important is to conduct a thermodynamic study, primarily the investigation of interactions between host and guest molecules with a goal of designing effective functional materials or systems for targeted bioapplications. Key aspects of host–guest behavior to be considered in this context are binding affinity and selectivity toward specific guest molecules, host–guest complementarity, selective and directional nature of intermolecular interactions, *etc.* Note that the supramolecular CB[*n*]-drug system possesses unique properties that can contribute to drug protection, stability, and its bioavailability. The first step of the molecular recognition process is unsurprisingly the inclusion of a drug within the hydrophobic cavity of cucurbit[*n*]urils which provides protection against environmental factors such as light, heat, oxygen, *etc.* The inclusion phenomenon enhances the solubility of the lipophilic drug in a water environment and can also prevent drug degradation. Maintaining drug stability over time can be accomplished by tailoring complexes of high stability. In the end, improved solubility and stability of drugs within the CB[*n*]-drug system can positively influence drug molecule's bioavailability. This is particularly important for drugs with low aqueous solubility where enhanced bioavailability can contribute to better therapeutic outcomes. Additionally, encapsulation of certain drugs within CB[*n*]s may reduce their toxicity by controlling their release and preventing excessive exposure to healthy tissues. Overall, interdisciplinary research studies of supramolecular principles of inclusion complexes in pharmacology significantly contribute to the development of advanced drug delivery systems, improved drug formulations, and enhanced therapeutic outcomes which makes this field very active and also challenging.^98,126^

**Fig. 12:**
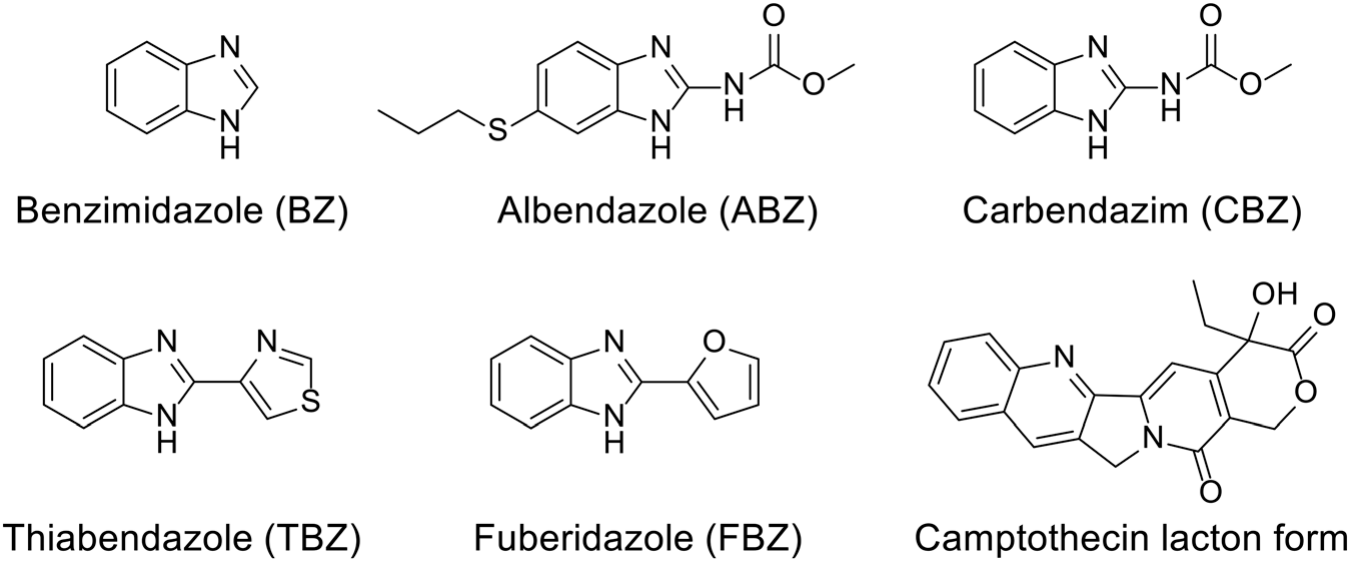
The studied benzimidazole drug series and camptothecin.

As already mentioned, CB[*n*]s are chemically and thermally exceptionally stable compounds that come in a variety of sizes and offer a broad range of cavity dimensions for potential pharmacophores. The only drawback of using CB[*n*]s as drug carriers or in biological applications in general is their poor solubility in water when compared to cyclodextrins or sulfonated calixarenes. However, with the optimization of functionalization methods^66,67^ or by changing the media, like adding biocompatible salts, or by changing the pH, CB[*n*] hosts can overcome their inherent solubility issues. On the other hand, these protocols often decrease complex stabilities due to the occurrence of competitive binding to the portals.^181^ Regarding the solubility of CB[*n*]s in various body fluids, it has been established that they are soluble in range of 1–4, 5–7, 34–45, and 33–37 mM in gastric,^182^ intestinal, nasal fluids and blood plasma, respectively.^143^ Moreover, mutual influence on solubility is established upon binding of the drug. Note that exploring modulation of chemical and biological properties of medically relevant guest molecules within a complex is crucial for designing CB[*n*]-based drug delivery systems with properties tailored to meet specific therapeutic requirements, as can be seen from the listed literature examples.^98,126^ In practice, this means that CB[*n*]s increase water solubility of the hydrophobic drug and *vice versa* encapsulation of already water soluble drug molecules can increase solubility of CB[*n*]s in a water environment.^98^ Increased solubility of drugs or bioactive compounds is achieved through host–guest interactions, as was observed for the complexation with either CB[*n*]s^98^ or acyclic-CB[*n*]s.^183^ Excellent examples of this are fungicides and anthelmintic drugs benzimidazole and its derivatives albendazole, carbendazim, thiabendazole, and fuberidazole which all have low solubility in water and lower affinities for CB[7] when unprotonated, traits that significantly diminish their use (Fig. 12).^184,185^ However, when these species are protonated, complex association constants become larger and preferential binding increases the p*K*_a_ values of the conjugate acids of these drug molecules by 2–4 units, consequently improving their solubilities.^184,185^ These complexes are therefore illustrative examples of the already mentioned p*K*_a_ induced complexation.^97–99^ Additionally, encapsulation in CB[7] also enhanced photostability of fuberidazole and thiabendazole,^184^ while the insoluble anticancer drug camptothecin upon encapsulation in CB[7] and CB[8] increased its solubility about 70 and 8 times, respectively.^186^ Lastly, encapsulation of a guest molecule into CB[*n*]'s cavities can even prevent thermal degradation of the guests.^187–189^

**Fig. 13:**
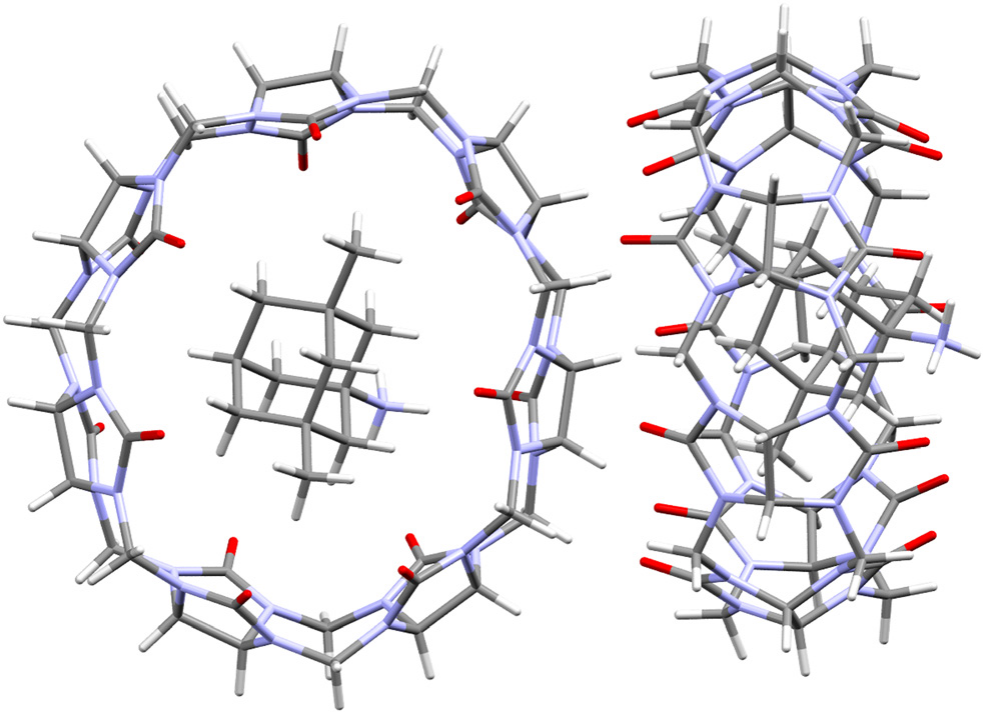
Single crystal X-ray diffraction structure (CCDC 746893) of the memantine hydrochloride/CB[7] complex.^187^

Wheate *et al.* examined the host–guest chemistry of four orally delivered drugs used in the treatment of human diseases, including also memantine, an Alzheimer's NMDA glutamate receptor drug.^187^ Although inclusion strength of memantine into CB[*n*]s was already determined^109^ and is compiled in Table 2, the authors undertook an exhaustive thermodynamic study of this system which included NMR and kinetic exchange data. Analysis of suitable monocrystals of the memantine hydrochloride/CB[7] revealed that memantine is fully bound within the CB[7] cavity and the complex is further stabilized by two hydrogen bonds (1.95 and 2.21 Å) between the guest's amine group and two separate carbonyls of the CB[7] portal (Fig. 13). This total encapsulation and strong interactions resulted in increased thermal stability of the supramolecular system, evidenced by a substantial melting point increase compared to the free drug (no melting observed below 380 °C).^187^ Prolongation of thermal stability has important implications for the prevention of drug degradation during its processing, formulation and storage.

Modulation of chemical and physical properties can improve drug stability since shielding in the cavity effectively prevents chemical reactivity (oxidation, hydrolysis), which is a common drawback that lowers drug bioavailability *in vivo*. This shielding effect is especially obvious in case of chemotherapeutic platinum-based drugs. Namely, it was observed that even partial encapsulation of a platinum drug within the cavity of a CB[*n*] can provide sufficient steric drug protection from nucleophilic attack from the compounds which are present in the human body in large amounts, like for example thiol peptides and proteins.^190,191^ Examples of drugs or biologically active compounds that have been studied in combination with CB[*n*] hosts are numerous and span a wide range of applications, for example, anaesthesia,^192,193^ treating infections,^188,194–197^ anticancer drugs,^164,186,189,190,198^ neuromuscular blocking agents (NMBAs),^199–201^ biodiagnostics,^202,203^*etc.*

### 3.3. Drug release

**Fig. 14:**
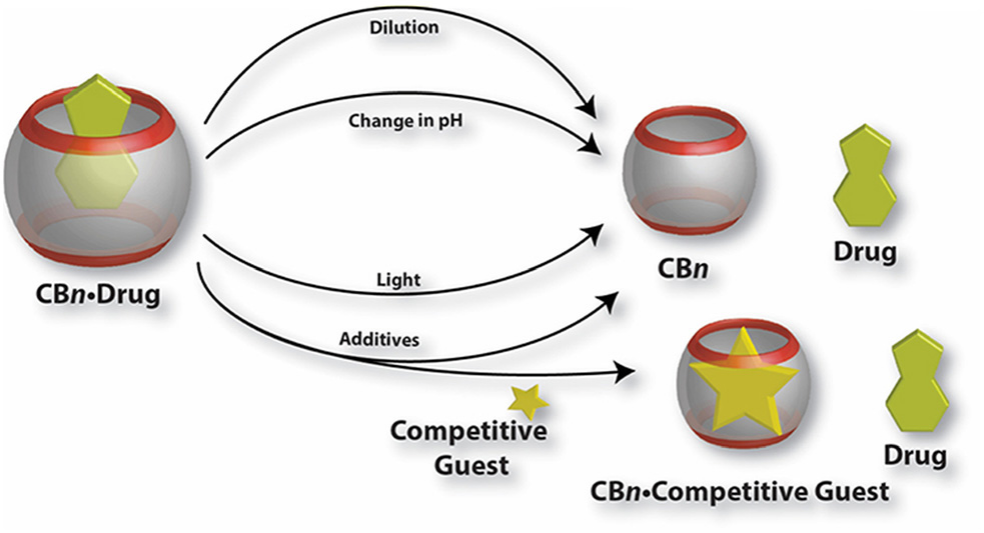
Illustration of various mechanisms for the release of drug molecules from drug/CB[*n*] complexes. Reproduced with permission from ref. 146, Frontiers Media SA, 2019.

Recall that an ideal drug delivery vehicle should not only have the ability to bind and protect a biologically active compound but it also has to overcome multiple biological barriers and finally release the drug molecule in a target cell, preferably in a slow and controlled manner.^204^ As illustrated in Fig. 14, drug release from a CB[*n*] cavity can usually be achieved by various methods which employ thermodynamic basis of interactions needed for complex formation. Since host and guest molecules create a complex with non-covalent interactions which are by nature weak and reversible, the real challenge of applying such a system is to achieve a balance between stable encapsulation and efficient release. In exceptional cases CB[*n*]s make complexes with host molecules with ultra-high affinity binding, but at a cost of very slow complex dissociation.^107,108,111^ In general, bioapplicable complexes are in the millimolar to micromolar affinity range, with dissociation rate constants of the order of seconds or faster. The dilution effect is a primary release mechanism of a biologically active compound happening soon after administration. It is known that the degree of complexation depends on the absolute concentrations of the host and the guest. Therefore, upon dilution the percentage of a complex will decrease as soon as the complex “enters” into a diluted region, *i.e.*, region with a smaller concentration of the complex.

**Fig. 15:**
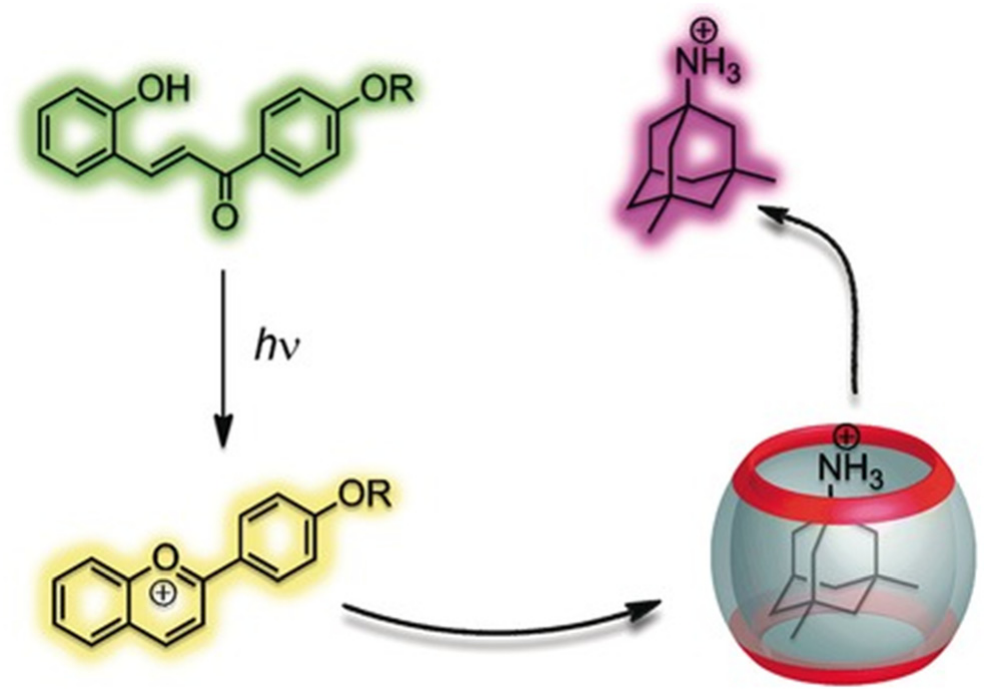
Controlling host–guest equilibrium with a flavylium-based photoswitch leads to efficient light-triggered release of memantine. Reproduced with permission from ref. 206, Wiley VCH, 2011.

Further mechanism that could be applied for drug release is a simple change of the pH of the environment because it can easily trigger drug decomplexation. The previously mentioned CB[*n*]-induced p*K*_a_ shifts^97–99^ can be used to trigger ejection of the molecule from the macrocyclic cavity, which is possible because CB[*n*]s have a much higher binding affinity for the protonated form of the drug when compared to its neutral form. Besides the influence of the pH of the biological medium, guest molecules can also be released by a phototriggered pH jump.^202,205–207^ A good example of that mechanism is a phototriggered reaction of a weakly binding neutral *trans*-chalcone that photochemically transforms into a flavylium cation, which is a strongly binding competitor and successfully displaces memantine from the CB[7] cavity in a water environment (Fig. 15).^206^ The authors also demonstrated that memantine can even be partially re-complexed if a thermally activated back reaction occurs, effectively reversing the supramolecular process.

**Fig. 16:**
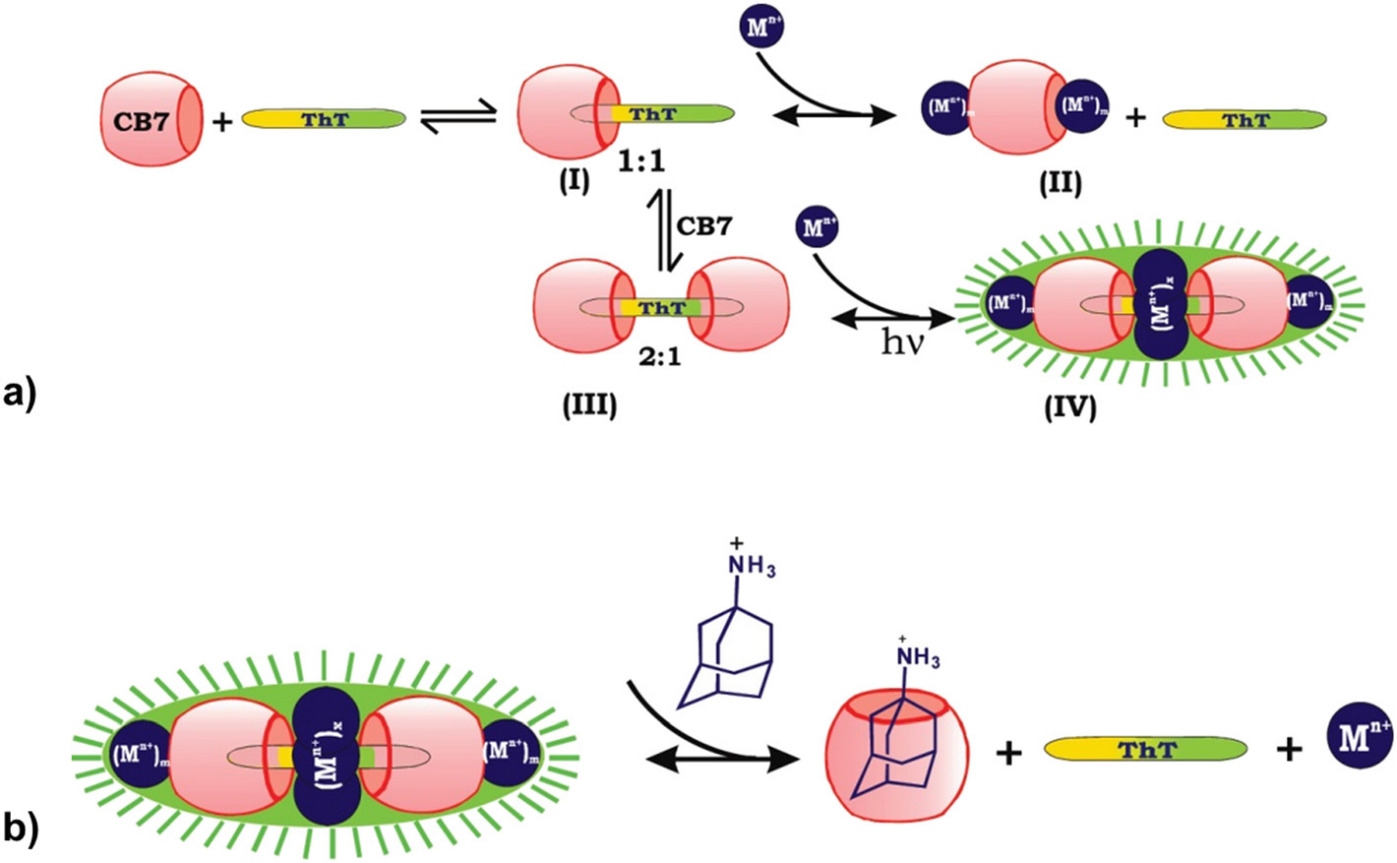
a) Binding interactions of the thioflavin ThT, CB[7] and metal ion system afford a highly fluorescent supramolecular capsule, (I) the 1 : 1 complex, (II) ion lids formation, (III) the 2 : 1 complex, and (IV) assembly of the cationic charges and sealing of the space between two CB[7] units. b) Release mechanism of ThT from the metal ion-bound supramolecular capsule by adding amantadine hydrochloride as a competitor. Reproduced with permission from ref. 209, the American Chemical Society, 2010.

Another way often used in releasing molecules captured in macrocyclic cavity is by competitive binding of inorganic cations^208^ to the portals which are already present in biological fluids or through displacement with another molecule that has a higher affinity for inclusion in the cavity. Bhasikuttan *et al.* demonstrated how precise control over the formation of the complex and release of the guest is challenging.^209^ They showed that thioflavin ThT, a fluorescent dye molecule used to diagnose neurodegenerative diseases, and CB[7] can yield two types of complexes with different host : guest stoichiometry, a 1 : 1 and a 2 : 1 complex (Fig. 16). Note that upon addition of the alkali or earth alkaline metal ions on the 1 : 1 and 2 : 1 systems different processes occur. In the 1 : 1 complex metal ions can bind to the host portals and thioflavin ThT is displaced from the cavity due to competitive binding. On the other hand, in the 2 : 1 complex the same metal ions serve the purpose of bridging vehicles between the two carbonyl rims of the macrocycles, forming a rather unusual assembly that is characterized by increased complex rigidity and stability, therefore causing its fluorescence. By addition of amantadine hydrochloride, an even stronger competitor, the fluorescent capsular complex is disassembled, thioflavin ThT released and a more stable amantadine/CB[7] complex is formed.^209,210^

**Fig. 17:**
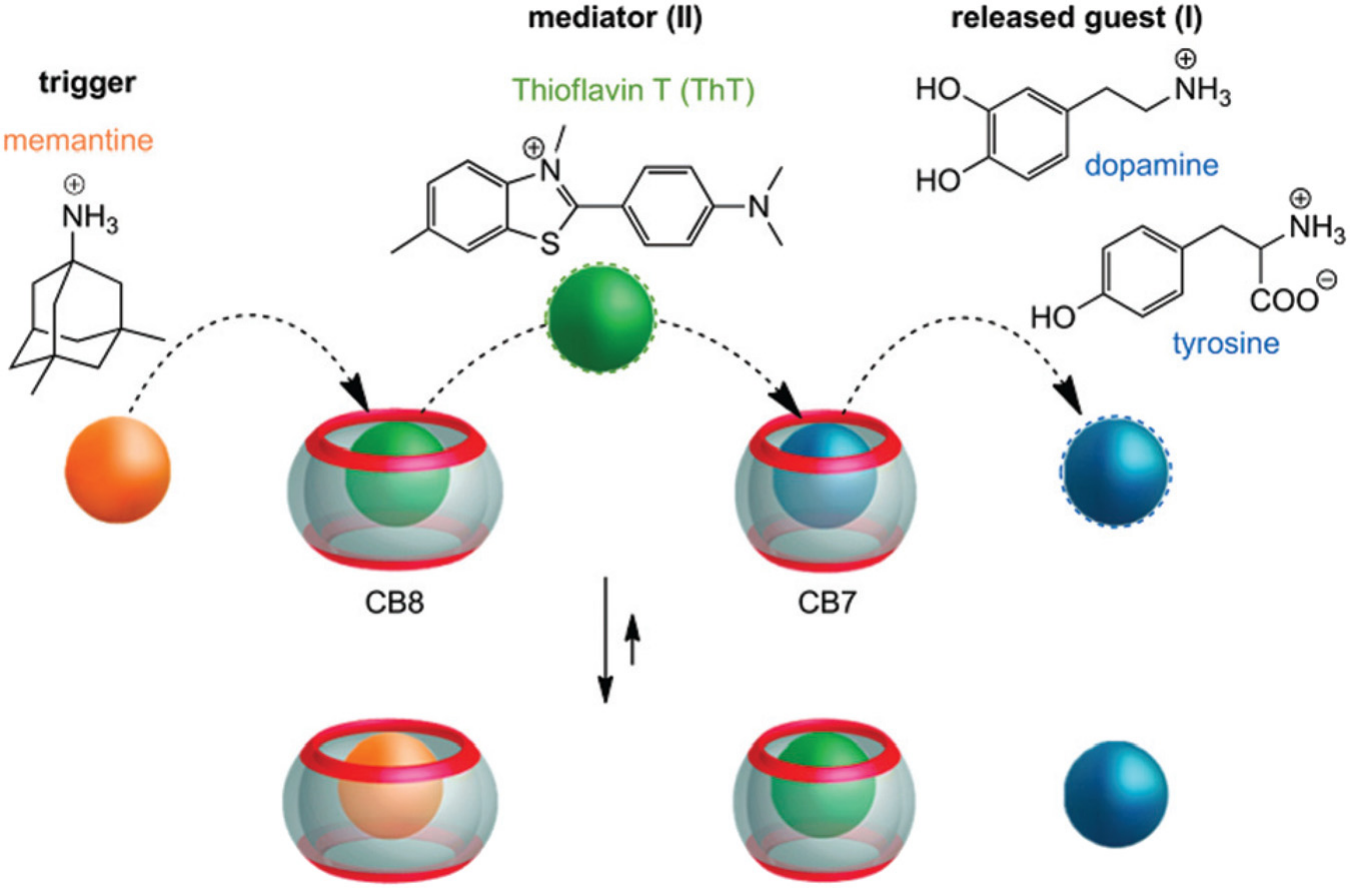
Chemically-triggered supramolecular communication cascade with memantine as the trigger, thioflavin ThT as the mediator and tyrosine or dopamine as the finally released guest. Reproduced with permission from ref. 211, the Royal Society of Chemistry, 2020.

Very recently, even multi-step competitive displacement chemically triggered by memantine was applied (Fig. 17).^211^ The demonstrated inter-complex reactions show some resemblance to biological systems and mimic how chemical information is communicated along a pathway in a cell. The cascade of displacements between three guest species and two CB[*n*]s is very well defined by thermodynamic characteristics of all involved host–guest complex equilibriums.^211^ In general, researchers often apply ADA derivatives as displacement guests in drug release processes,^209,210,212–218^ especially amantadine or memantine, that are in turn also well-known drugs for viral infections, Alzheimer's disease, *etc.*^10^ This feature is especially attractive when using drug targeting to produce a combined activity from two drug species.

On another note, supramolecular approach to PEGylation (covalent conjugation of polyethylene glycol) was used in controlling the insulin release *vs.* standardized covalent PEGylation in which the protein in question is permanently modified and thus can have reduced activity and increased immunogenicity. Namely, as a proof-of-principle it was shown that by varying different host–guest affinities it is possible to tune the pharmacokinetic profile of the protein in question. Again, the ADA/CB[7] system was chosen because of its excellent binding strength and the complex was thus associated from the ADA scaffold bound to PEG_20k_ and CB[7]-modified insulin.^219^

### 3.4. Bioapplication of ADA/CB[*n*]

Supramolecular regulation of intracellular events or tuning of biological functions in which supramolecular systems cooperate to some extent has potential future application in biological systems. Adamantane and cucurbit[*n*]uril molecules as well as their subsequent supramolecular assemblies have the potential to modulate various cellular functions and they are not limited only to the most commonly used drug delivery and controlled release applications. Survival and functionality of supramolecular complexes within a cellular environment are critical considerations for their application in various biomedical contexts, as can be seen from the examples herein, with ADA/CB[*n*] having a great advantage due to its inherently high stability constant. The real challenges for supramolecular complexes chosen to be exploited in living cell are not only to fulfill the basic requirements of biocompatibility, stability, resistance to degradation and efficient cellular uptake but also to preserve their endurance and function within the complex in a dynamic cellular environment. Assemblies designed in a specific manner in order to reliably interact with each other can modulate signaling pathways and cellular responses or influence intracellular signaling cascades to regulate cellular functions and processes. Incorporation of imaging agents into the ADA/CB[*n*] system, *e.g.*, fluorescent probes, for cellular imaging purposes can help in tracking and visualization of the assembly within cells. Additionally, using the assembly for diagnostic imaging enables identification of specific cellular markers or abnormalities in tissues. Previously discussed examples showed how designed assemblies can respond to specific cellular stimuli (*e.g.*, pH, temperature, enzymatic activity), allowing for a dynamic and responsive behavior within the cellular microenvironment. Researchers in this field aim to design complexes that can precisely interact with cellular components, providing a platform for therapeutic interventions, diagnostics and understanding of fundamental cellular processes.

**Fig. 18:**
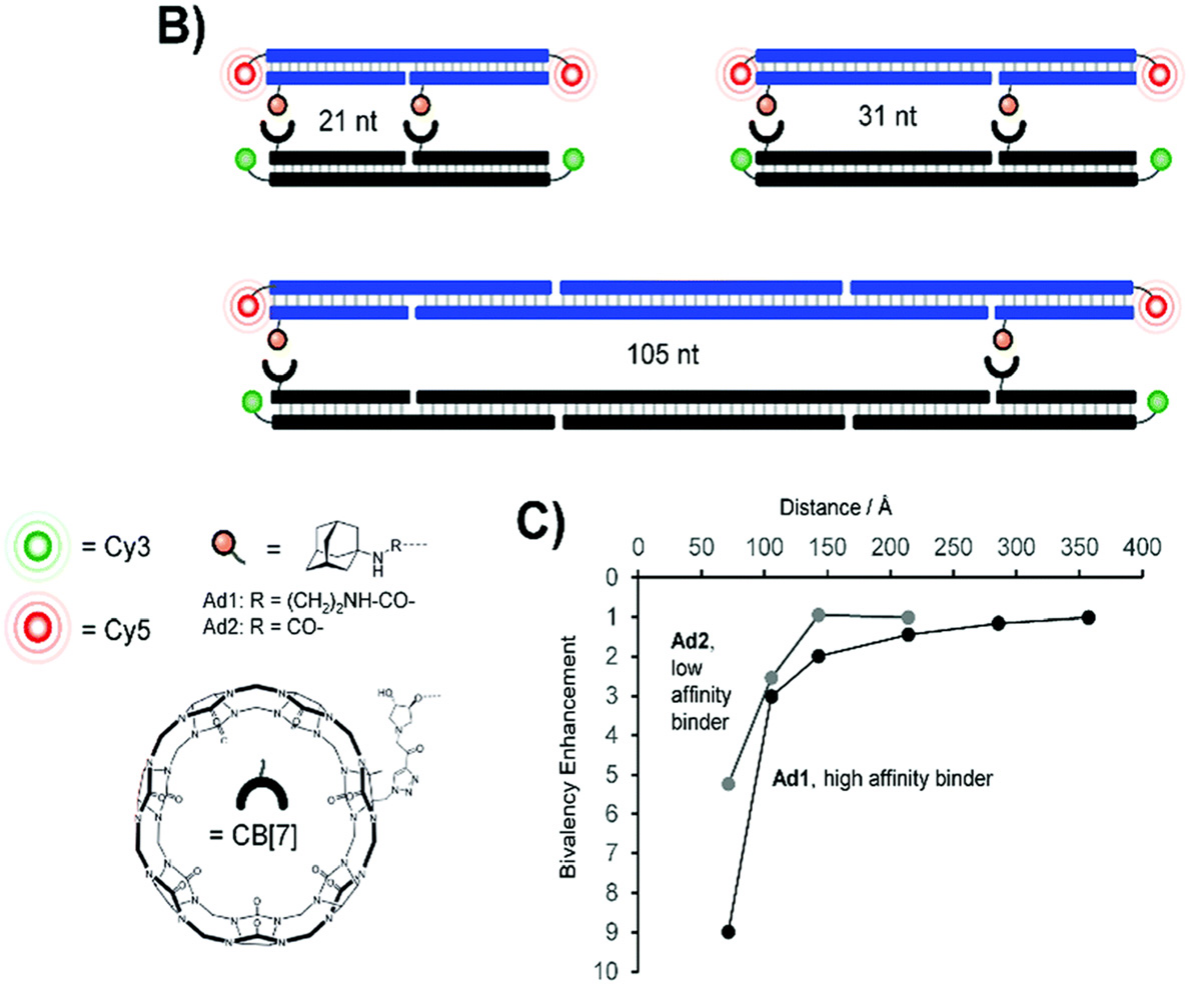
Bivalent library with separated ADA/CB[7] host–guest pairs (top, B) and distance dependency of bivalency-enhanced interactions between distance-matched CB[7] and ADA displays (bottom, C). Reproduced with permission from ref. 222, the Royal Society of Chemistry, 2020.

Protein–protein interactions are fundamental to the functioning of cells, regulating diverse important physiological functions at a cellular level and playing a crucial role in signal transduction, gene expression, cellular structure, and other essential functions. Protein recognition processes can occur throughout multivalent engagement between separated binding sites that can even cooperate in a synergistic manner and thus induce enhancements of overall binding affinity. Reliable and efficient host–guest interactions between CB[7] and ADA serve as a model system to test whether multivalent interactions will still occur at larger distances between the binding sites, thereby contributing to the intricate network of cellular pathways, and help unravel fundamental cellular processes.^220^ For that purpose stable and long DNA scaffolds with two attached CB[7] units and two various ADA ligands at the distances varied from 70 to 360 Å were used in order to explore the distance-stability relationship and its limitations in complexes formed upon bivalent interaction (Fig. 18).^220,221^ The scaffolds were additionally fluorescence-labeled to assess the interactions (cross-linking) between CB[7] and the ADA moieties. It was found that the affinity gain provided by bivalency is indeed dependent on and controlled by the strength of individual monovalent interactions, by the distance between host–guest pairs and the overall flexibility of the scaffold. This suggests that binding systems of low affinity and distant sites will be more likely cross-linked *via* long flexible tethers, thereby enabling a nearing of the respective DNA strands.^220^

**Fig. 19:**
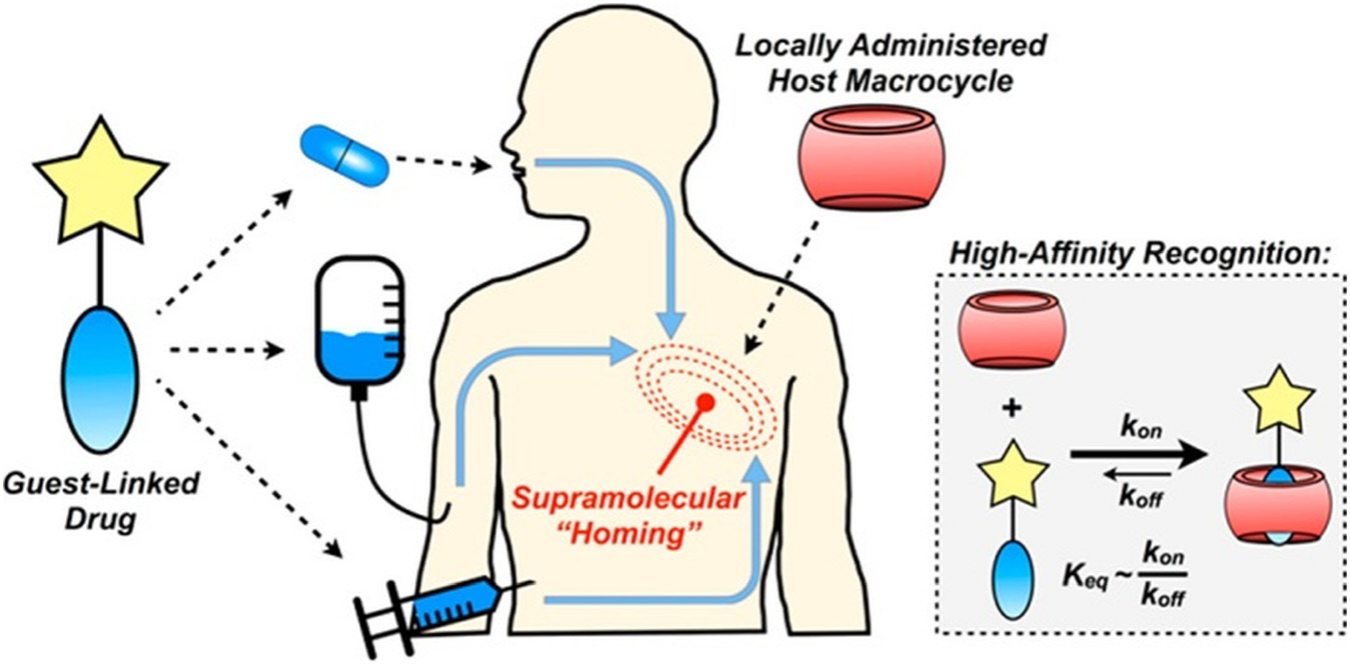
Supramolecular homing of guest-appended small molecules on the basis of affinity for locally applied host macrocycles. Reproduced with permission from ref. 223, the American Chemical Society, 2019.

A recent report details how host–guest chemistry was employed in locally administrated targeted drug delivery.^223^ The idea was that a CB[7]-rich injectable hydrogel would be applied into the tumor tissue and would be used to facilitate drug localization through high affinity that would lead to substantial drug aggregation at the target site (Fig. 19). In order to validate the effectiveness of this strategy, the authors first synthesized test molecules which consisted of the anchoring group for the CB[*n*] cavity and a fluorophore replacing the drug at its defined position in the compound. The ADA/CB[7] system was used as a model of a complex with moderate association constant while ferrocene/CB[7] was labelled as a strong guest complexing agent. After repeated administrations, fluorescence in the target site increased linearly, suggesting that the proposed strategy was indeed efficient. However, since the ADA/CB[7] system had lower targeting loading than the corresponding ferrocene, the latter was chosen for the *in vivo* experiments in combination with the drug doxorubicin. Nevertheless, these findings illustrated that substance aggregation in tumors was significant, which has implications for possible higher dosing of active species at the tumor site without potential concomitant issues from off-site toxicity.

**Fig. 20:**
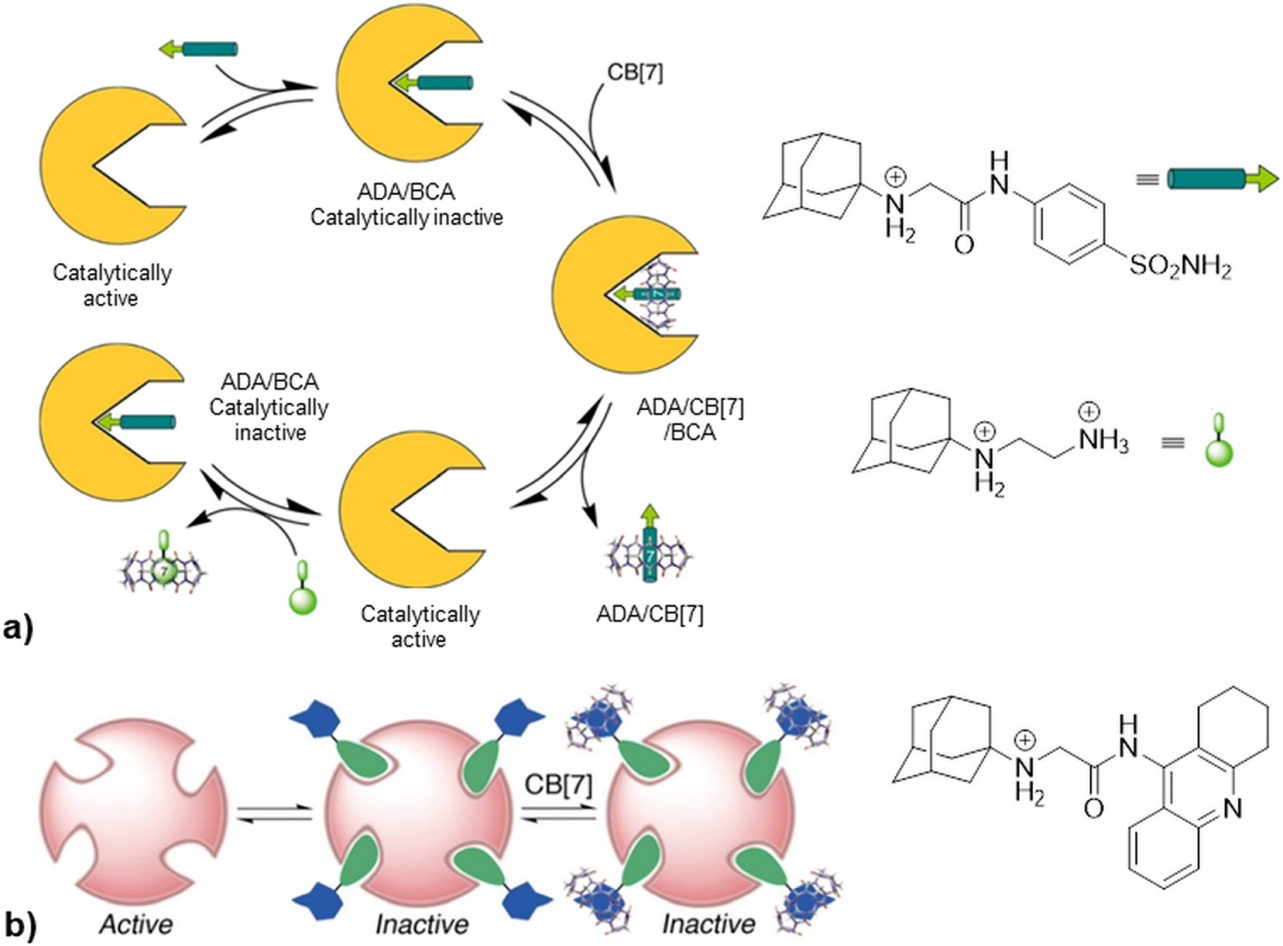
a) Representation of the control of enzymatic activity of BCA based on enzyme and CB[7] competition for common inhibitor, b) formation of ternary complex upon addition of CB[7] to ADA/AChE. Reproduced with permission from ref. 224, the American Chemical Society, 2010.

Going further, in the next example authors take advantage of non-covalent interactions between CB[7] and synthetic amine derivatives that exceed biological macromolecule affinity for their targets by taking control over the enzymatic activity of bovine carbonic anhydrase (BCA) or acetylcholinesterase (AChE).^224^ Synthesized inhibitors consisted of two binding regions, an enzyme-binding group and a macrocycle-binding group. The first domain contained a benzenesulfonamide group which is a known inhibitor scaffold responsible for binding into the enzyme active site and the second domain contained 1-aminoadamantane, trimethylsilylmethyl amine or hexylamine responsible for docking into the CB[*n*] cavity. As shown in Fig. 20, addition of CB[7] to a catalytically inactive form amine/BCA afforded a ternary transient complex amine/CB[7]/BCA that underwent rapid dissociation to a free enzyme, which thereby restored the activity along with producing a supramolecular complex of the synthesized inhibitor in a CB[7] container, amine/CB[7]. By adding a strong competitor (the ADA guest derivative) into the systems, the inhibitor molecule is displaced from the CB[7] and thus regenerated to settle again into the active site of the enzyme, which is in turn inhibited again. The authors state that on–off switching of the catalytic activity can be repeated in several cycles and can be monitored by combined spectroscopic techniques such as UV/vis, fluorescence and ^1^H NMR. On the other hand, upon addition of CB[7] to the inhibited AChE no recovery of enzyme activity was observed. A possible explanation of that finding could be that relatively open active and peripheral sites of AChE can accommodate both inhibitors and their CB[*n*] complex, as shown in Fig. 20. Competitive binding of the two-faced inhibitors with enzyme *vs.* CB[7] was thus used as a controlling factor for enzyme activity. The authors highlight that this principle is different from typical allosteric regulation of enzyme activity found in nature where a small molecule typically binds to a site distant from the enzyme's active site with a resulting decrease or increase of enzymatic activity.

**Fig. 21:**
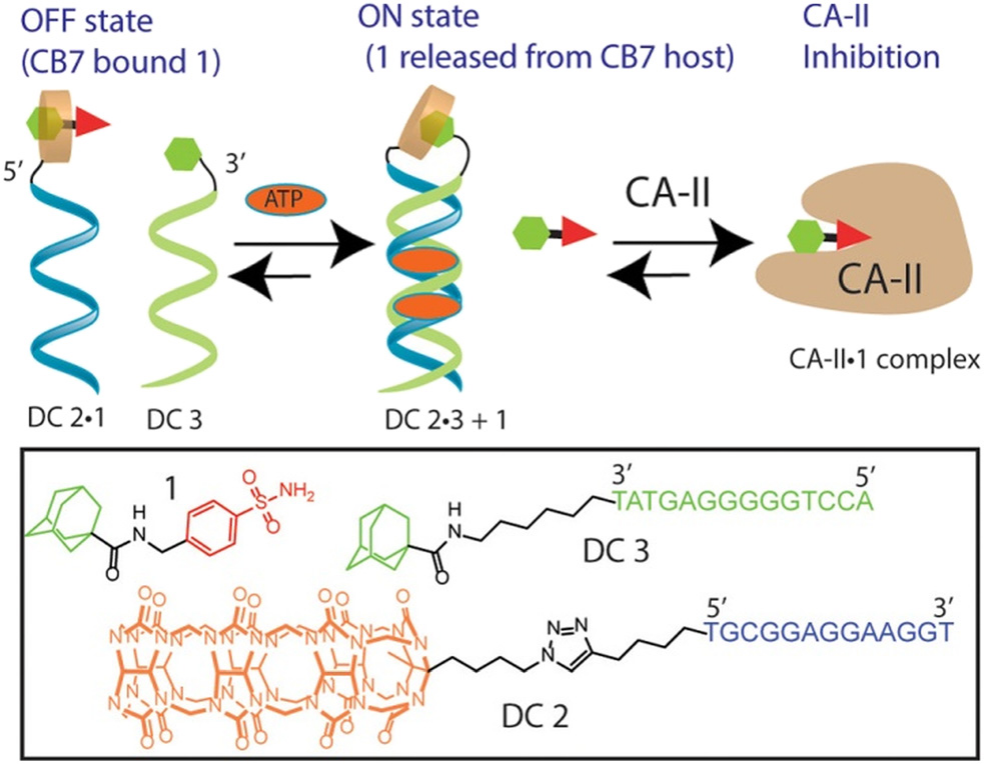
A DNA-small molecule chimera (DC) transducer based on a split DNA aptamer that converts an ATP input into functional release of a CA-II inhibitor ADA from the CB[7] host. Reproduced with permission from ref. 225, the American Chemical Society, 2017.

Host–guest recognition between adamantane and cucurbit[*n*]uril offers a versatile platform for designing DNA-based systems with enhanced functionalities. Here we will mention several examples in which the ADA/CB[7] system can be employed in conjunction with DNA to control activities of enzymes within cells, to regulate and intervene in DNA processes, *etc.* The CB[*n*] macrocycle was recently, for the first time, covalently linked to a DNA-small molecule chimera while the ADA scaffold was linked to another DNA chain with the aim to use the emerging host–guest complexes to control the activity of carbonic anhydrase II (CAII) (Fig. 21).^225^ Adenosine 5′-triphosphate (ATP) was used as a trigger that led to the displacement of a protein inhibitor guest molecule bound within a CB[7] host. The event was accomplished by combination of the ATP-induced DNA duplex assembly and intramolecular host–guest interactions. Note that once again the strong interactions of the supramolecular ADA/CB[7] complex are used to control the inhibition of CA-II.

**Fig. 22:**
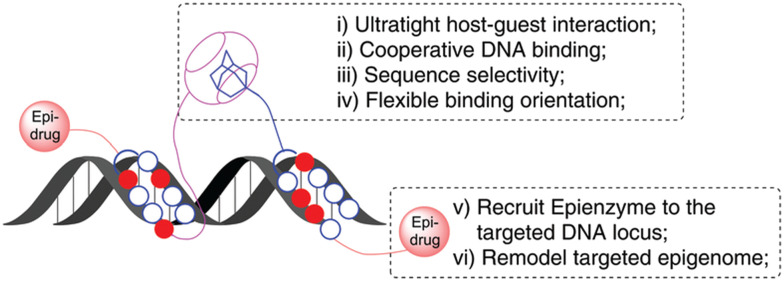
Illustration of an ADA/CB[7] assisted epigenetically active DNA binding system. Reproduced with permission from ref. 226, the Royal Society of Chemistry, 2020.

Similarly, in 2020 a group of authors reported on an ADA/CB[7] assisted epigenetically active DNA binding system that mimics transcription factors pair and recruits the epigenetic modifier to a particular DNA target site (Fig. 22).^226^ The approach of the host–guest system is believed to happen first, followed by synergic DNA binding.

**Fig. 23:**
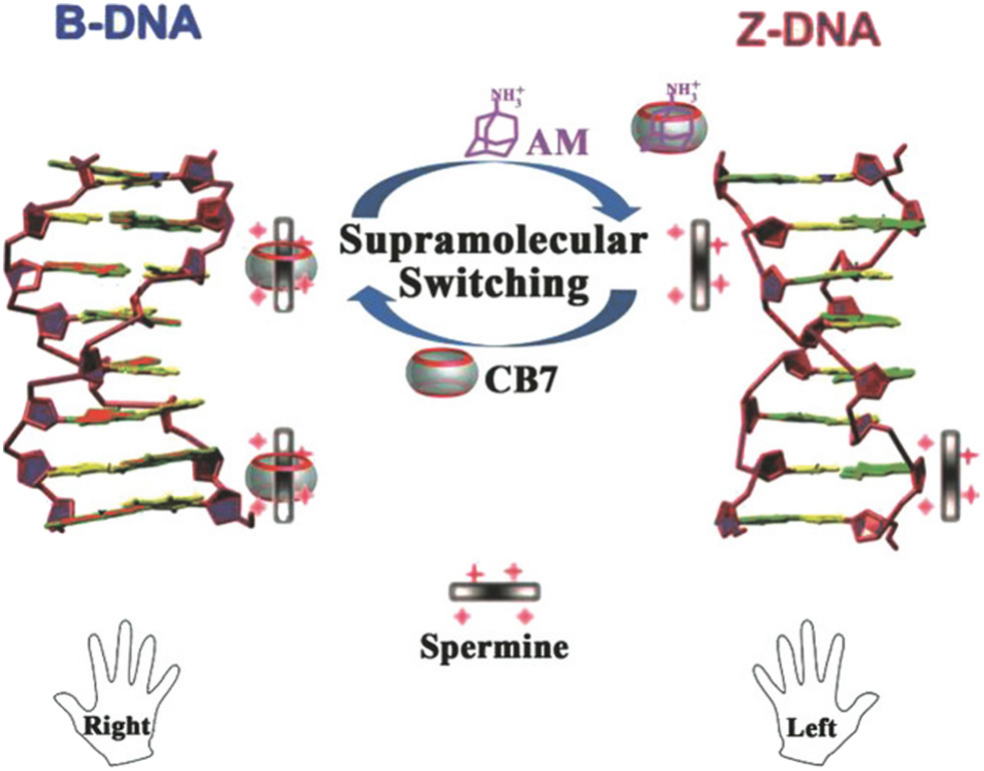
A CB[7]-based supramolecular approach for reversible B/Z-DNA transition. Reproduced with permission from ref. 227, Wiley VCH, 2018.

Employment of CB[7], spermine, and 1-aminoadamantane has recently been studied as a supramolecular platform for the regulation of a DNA transition process, as shown in Fig. 23.^227^ Right-handed helical DNA (B-DNA), which is more favored and thermodynamically more stable, could be converted into left-handed helical DNA (Z-DNA) in the presence of spermine. This transition process could be reversed upon addition of CB[7] which binds spermine and thus induces a conversion of Z-DNA to B-DNA. Reversely, active spermine could be again released upon addition of 1-aminoadamantane which is a CB[7] binding competitor and therefore readily induces reconversion of the B- into the Z-DNA helical structure. Additionally, the authors noted that a cycled transition could be achieved by repeatedly adding CB[7] and 1-aminoadamantane.

**Fig. 24:**
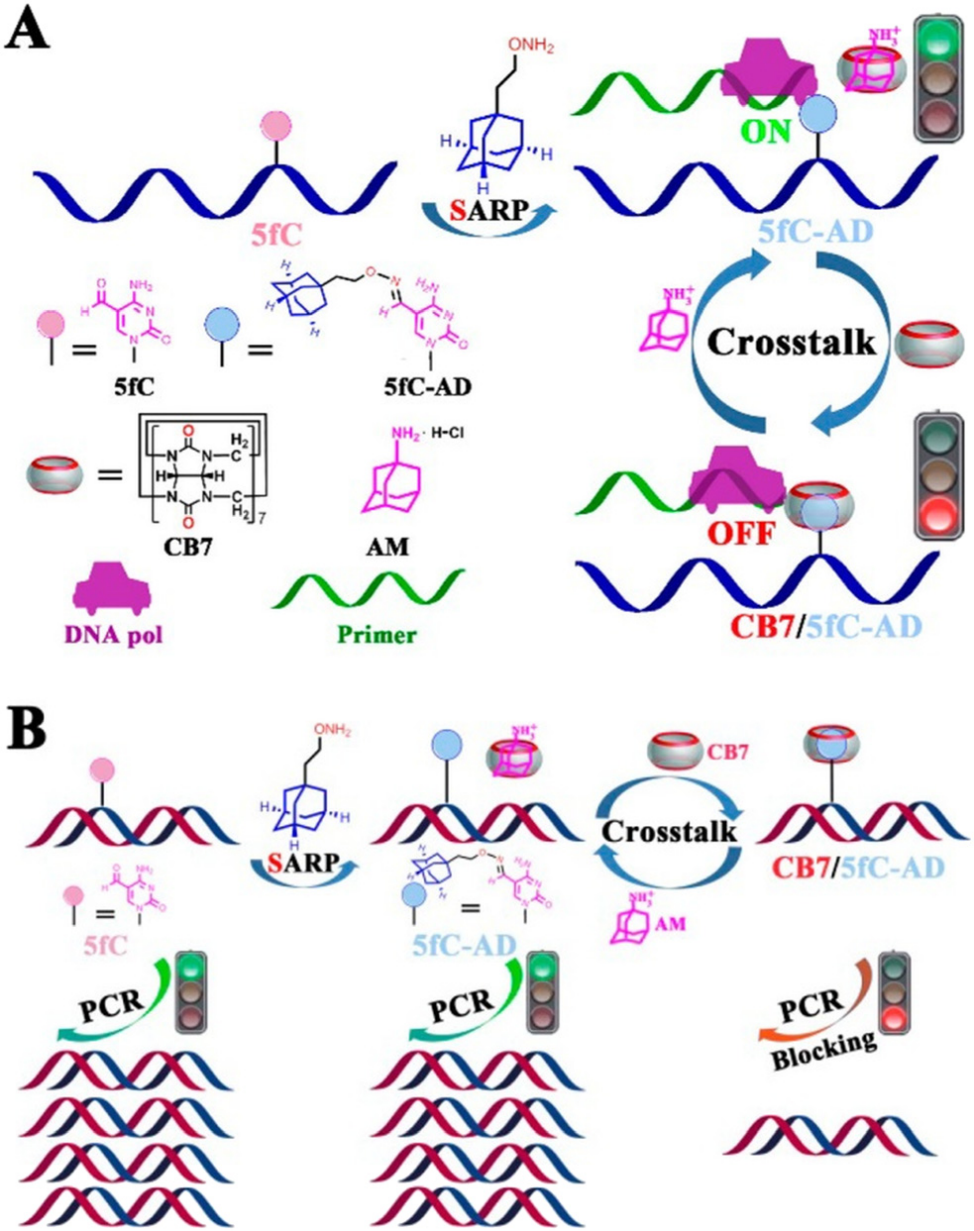
Reversible intervention of 5fC-targeted DNA pol elongation and PCR reaction (A and B). The binding of CB[7] to 5fC-AD nucleotide is assumed to generate large steric hindrance that prevents the approach of DNA pol and inhibits enzyme activity. The CB[7]/5fC-AD host–guest interactions can be reversed by treatment with 1-aminoadamantane. Reproduced with permission from ref. 228, the American Chemical Society, 2017.

In the next example DNA was modified with ADA-functionalized 5-formylcytosine (5fc-AD) which served as a binding anchor for the CB[7] cavity.^228^ By utilizing supramolecular interactions of the ADA/CB[7] system it was possible to reversibly intervene in biochemical activities of the DNA, including restriction endonuclease digestion, DNA polymerase elongation, and polymerase chain reaction. It was also demonstrated that steric hindrance of the rigid CB[7] prevents the enzyme from binding to the substrate, whereas the CB[7]/5fC-AD host–guest interactions can be reversed by treatment of the system with a competitor substrate like 1-aminoadamantane (Fig. 24).

Jayawickramarajah and coworkers broadened the toolbox of dynamic DNA chemistry and nanotechnology by introducing a synthetic surrogate and utilizing ADA/CB[7] interactions for DNA strand displacement. A classical concept of base-pair-driven toehold-mediated strand displacement (BP-TMSD) was replaced with host–guest-driven toehold-mediated strand displacement (HG-TMSD), which showed possibilities for enzyme activity control and layered reactions that detect specific microRNA.^229^

**Fig. 25:**
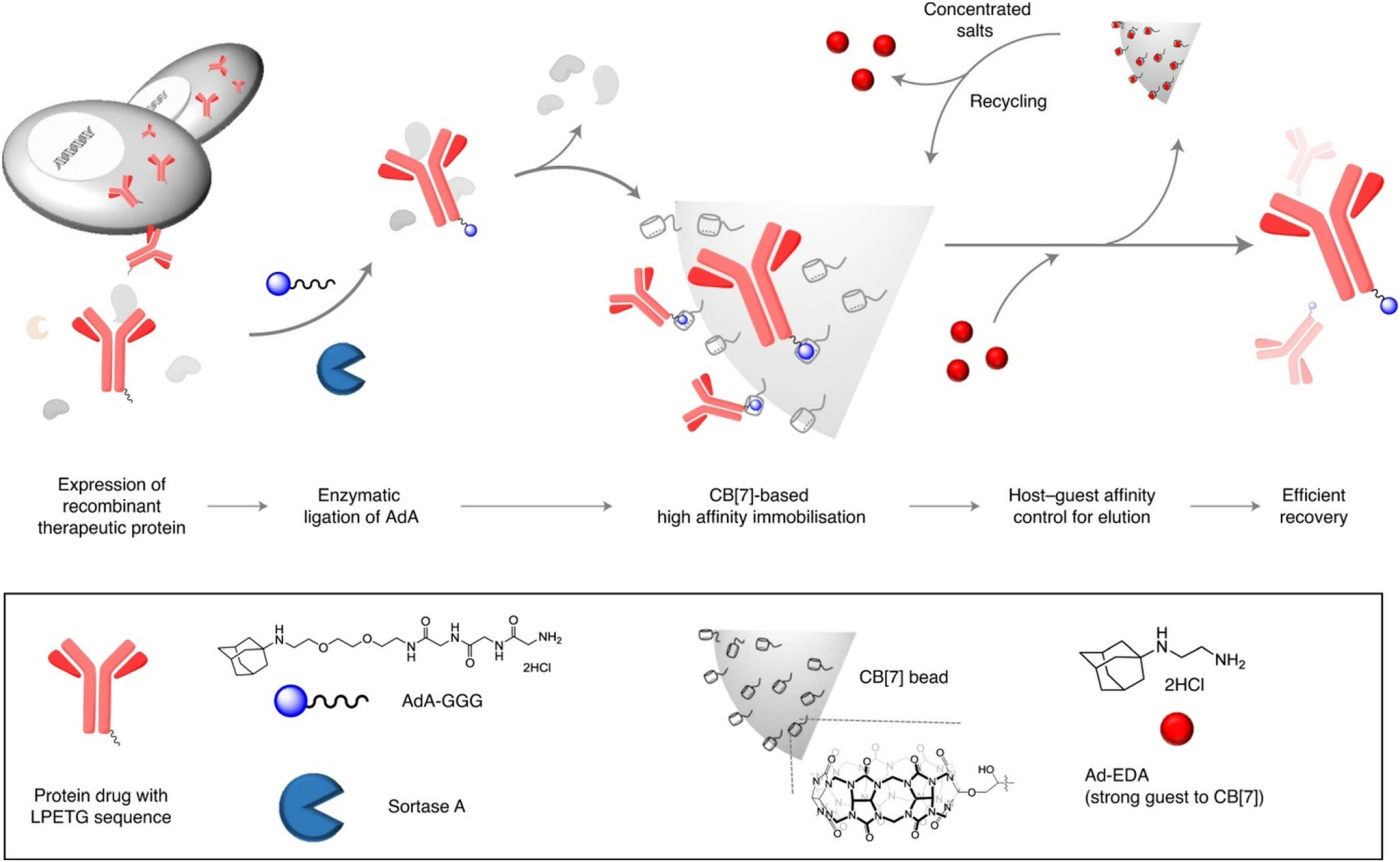
Recombinant therapeutic proteins with the LPETG sequence expressed in cells are tagged with ADA–GGG *via* sortase A ligation and the tagged proteins are then selectively captured on CB[7]-conjugated beads and efficiently recovered *via* strong guests. Reproduced with permission from ref. 230, Nature Research, 2020.

Efficient protein purification can be laborious work and has many drawbacks such as protein denaturation, contamination, low scale, *etc.* Kim's group recently proposed a scalable method for affinity purification of large quantities of recombinant therapeutic proteins with the aim of utilizing supramolecular interactions of an ADA/CB[7] system (Fig. 25).^230^ Herceptin, an antibody drug used to treat breast cancer, was first site-specifically tagged with 1-aminoadamantane *via* an enzymatic reaction. A highly specific and selective inclusion of the ADA scaffold into the CB[7] cavity was used for binding with the CB[7]-conjugated agarose beads. Lastly, the protein was recovered in high purity using a competitive guest affinity for binding into the CB[7]. The stable and robust CB[7]-beads can be recycled up to 4 times by treatment with concentrated salts. Here it should also be mentioned that quite recently a new strategy of a ADA/CB[7] protein assembly for affinity purification of methyllysine proteomes has been successfully employed.^231^

**Fig. 26:**
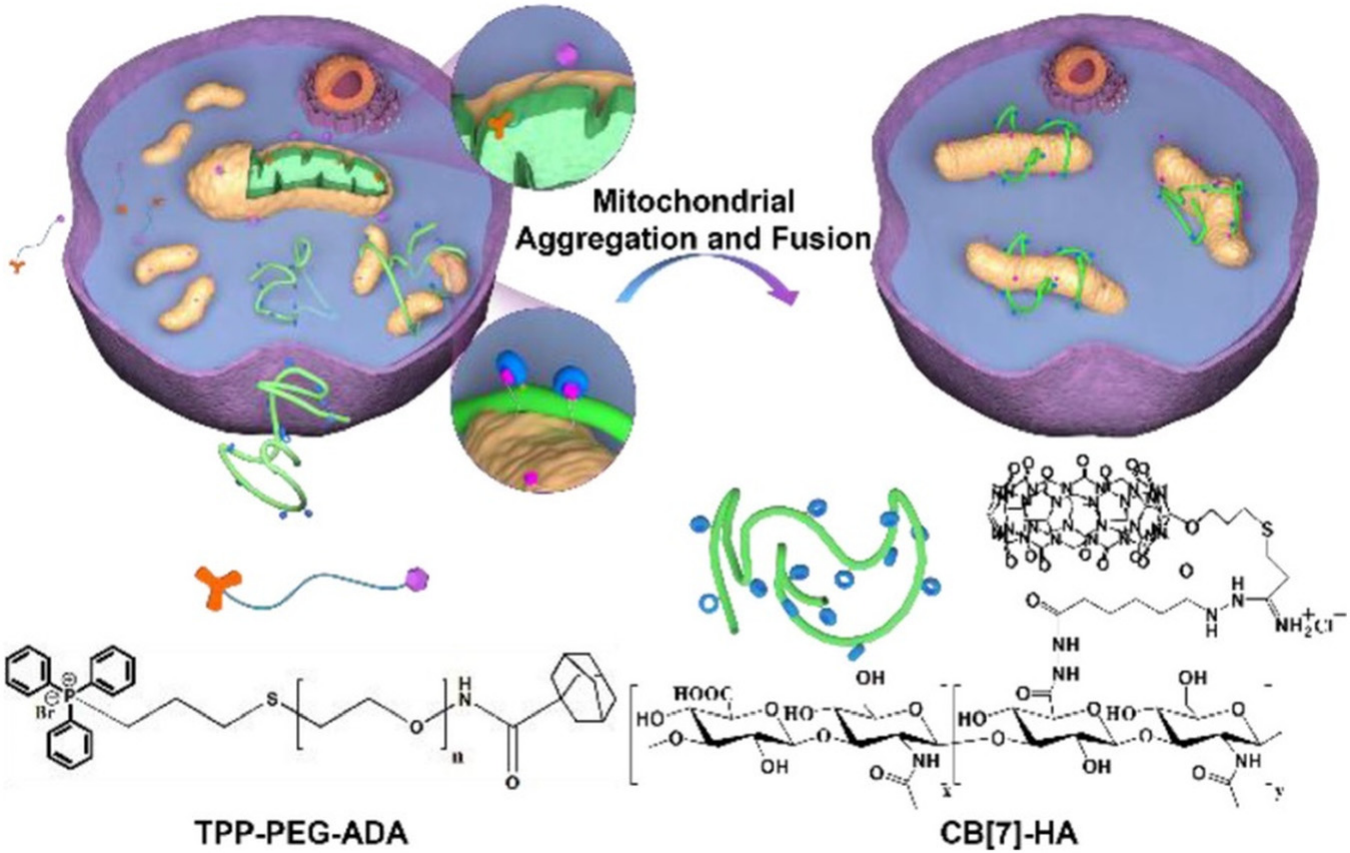
Illustration of supramolecular mitochondrial aggregation and fusion. Reproduced with permission from ref. 232, American Chemical Society, 2020.

Development of oxidative stress related diseases is often associated with the mitochondrial fission, since the fragmentation of mitochondria membrane advances production of reactive oxygen species and promotes apoptosis. The first example of an artificial pathway to induce mitochondrial fusion *via* supramolecular mitochondrial aggregation was proposed to be the inception of a completely different approach when compared to traditional methods of fusing mitochondria by manipulating specific proteins.^232^ Namely, employment of ADA/CB[*n*] system in engineering living cells is possible because of its precise design where the puzzle pieces interact specifically with each other and allow for a precise control over cellular processes. First, mitochondria are surface-modified with a guest species, *i.e.*, adamantane along with polyethylene glycol (PEG) system that was tagged with triphenylphosphonium (TPP) (Fig. 26). Afterwards the addition of CB[7] modified with hyaluronic acid (HA) induced supramolecular aggregation and subsequent fusion of mitochondria *via* ADA/CB[*n*] strong host–guest interactions that were employed to cross-link several mitochondria together. It should be noted that the chemically stressed SH-SY5Y cells and zebrafish neurons were effectively protected when this supramolecular mitochondrial fusion strategy was applied *in vitro* and *in vivo*, respectively, suggesting that the described approach may be applicable in a broader sense in the future.

**Fig. 27:**
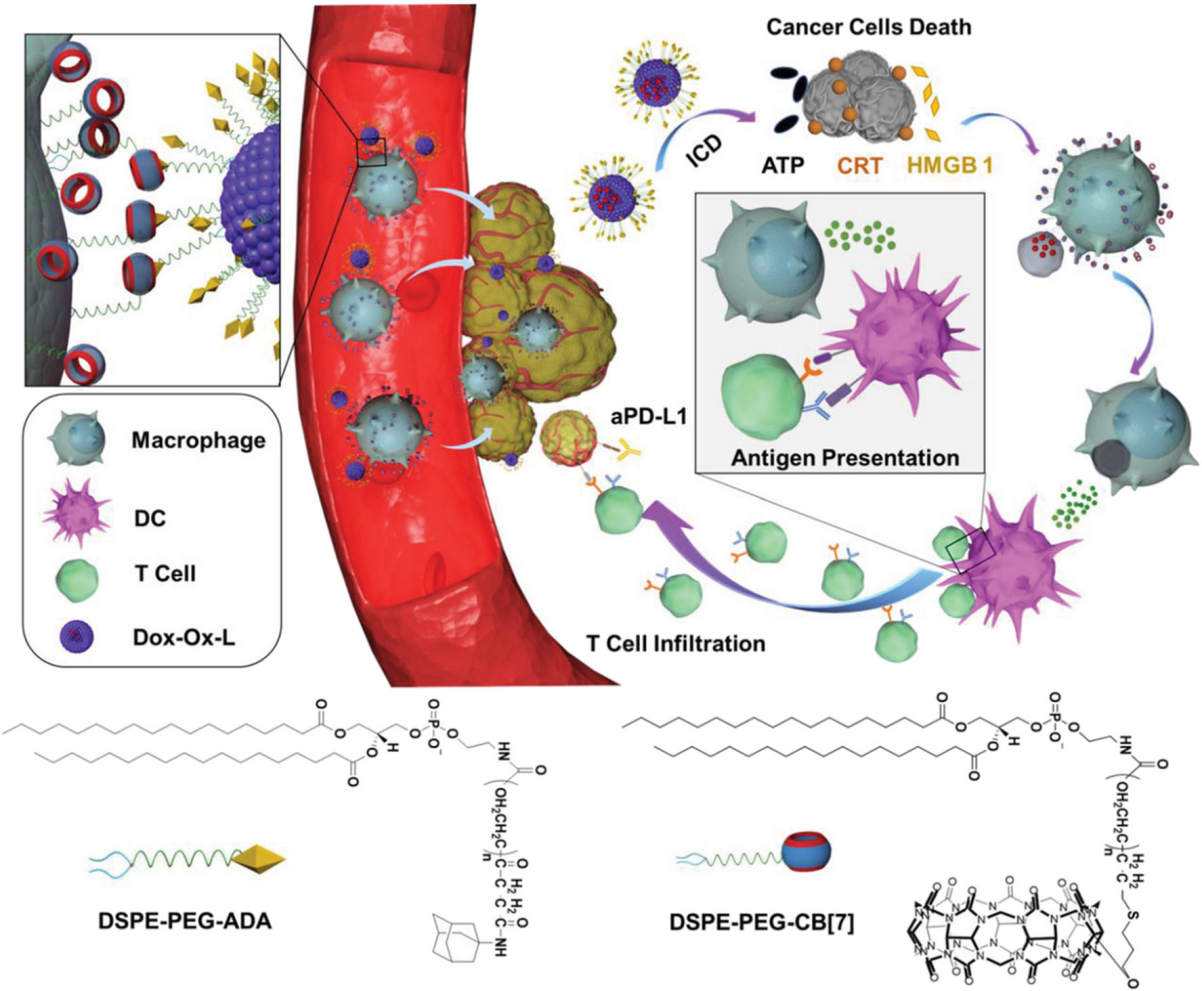
Illustration of a cell-hitchhiking drug delivery enabled by host–guest interactions between CB[7] modified macrophage and ADA modified liposome, leading to melanoma cell death. Reproduced with permission from ref. 233, Wiley VCH, 2021.

Furthermore, by functionalizing the ADA/CB[*n*] assembly with ligands for specific cell types one can enable targeted interactions or by tailoring the assembly for disease-specific markers achieve targeted therapy. Recently, Wang and coworkers demonstrated that supramolecular recognition can be used to anchor drug-loading liposomes (L) on macrophages (M) for cell-hitchhiking drug delivery where “hand-holding” and “marriage” between CB[7] and ADA functionalized on the surface of the macrophage and liposome, respectively, was the driving force to obtain the M–L conjugate (Fig. 27). It was found that the resulting complex remained stable for up to at least 4 h, which was sufficient for *in vivo* hitchhiking delivery of toxic drugs loaded in a liposome to the tumor, consequently enhancing the chemoimmunotherapy of melanoma in mice. The authors concluded that the CB[*n*] and ADA systems which are stable for months could have potential in clinical translation, for example in a form of a biological kit consisting of drug-loaded ADA liposomes and CB[7] functionalized moieties present as two separate reagents.^233^

Research in this area is dynamic and advancements in supramolecular chemistry are continually expanding the possibilities for utilizing assemblies like tightly bound ADA/CB[*n*] complexes in cellular and organismal regulation. However, it is essential to approach these applications with a thorough understanding of cellular processes, biocompatibility and safety considerations.

### 3.5. Bioanalytics of ADA/CB[*n*]

**Fig. 28:**
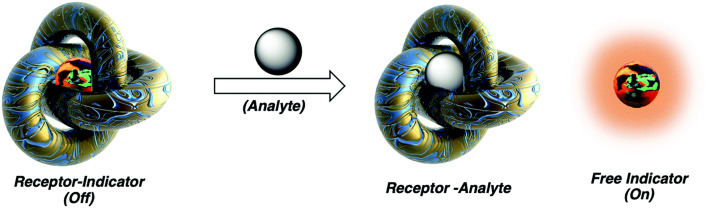
Analyte mediated displacement of an indicator from a supramolecular host. Reproduced with permission from ref. 234, Royal Society of Chemistry, 2021.

Chemosensing in biological systems, especially physiologically relevant ones, is highly challenging due to nanomolar to micromolar concentration range of the analyte of interest and also due to interference of highly competitive substrates present in such environment. Cucurbit[*n*]uril host–guest complexes have also emerged as promising tools for biologically relevant analytical^135–137^ and diagnostic applications, owing to their stable and specific interactions, bio-orthogonality in binding, refined synthesis, thermodynamic stability, *etc.*, but most importantly due to their high selectivity and strong binding. CB[*n*]-based molecular sensors have lately been employed as indicator displacement assays (IDAs)^234^ for detection of various ADA derivatives in biological environmental conditions, *e.g.*, for acetyl amantadine^235^ or amantadine^236^ in urine, amantadine in human serum^237,238^ and memantine in blood serum^239^ or in biofluids.^240^ Unlike direct binding assays (DBA) where binding of an analyte to the CB[*n*] cavity yields a spectroscopic response but is not very selective, in IDAs the CB[*n*] cavity is pre-complexed with a fluorophore or a chromophore molecule which is upon analyst binding displaced and shows observable specific response (Fig. 28). In continuation selected examples of IDAs with ADA/CB[*n*] systems where the ADA derivative is a drug or a metabolism product will be presented.

**Fig. 29:**
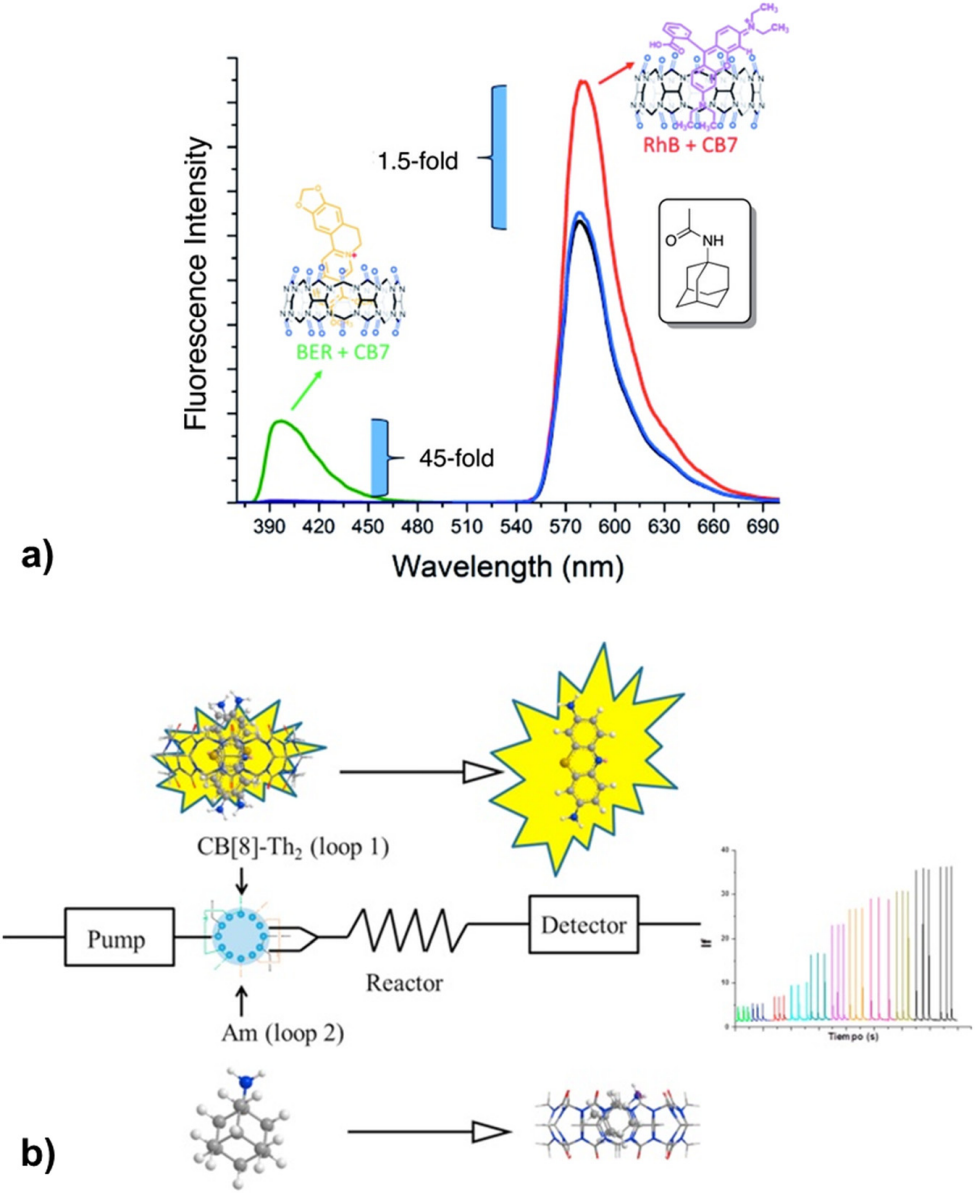
a) Displacement of rhodamine B (RhB) and berberine (BER) from CB[7] during fluorescence titration with acetyl amantadine (AcAm) in deionized H_2_O and b) amantadine determination in human serum using a fluorometric flow injection analysis system. Reproduced with permissions from ref. 235, Canadian Science Publishing, 2016 and from ref. 237, Elsevier, 2018.

Recently it was proposed that analysis of acetyl amantadine (AcAm) in urine could serve as a diagnostic tool to measure the spermidine/spermine *N*^1^-acetyltransferase (SSAT) activity.^234^ The ADA derivative in question is exclusively a product of the metabolism of amantadine (Am) upon catalysis with SSAT, which in turn is known as a biomarker for multiple aggressive cancers. The authors used the IDA detection concept of fluorescent indicators bound to CB[*n*] that was afterwards replaced with AcAm due to the ADA/CB[*n*] system's capability for strong and selective binding (Fig. 29a). The limit of detection of AcAm was 0.087 μM with a linear response range from 0 to 1 μM. This study is therefore a proof-of-concept for designing similar analytical testing methods, however, since quantitative sensing was not achieved it is not yet of clinical relevance.

In another approach, a competitive assay between the thionine dye and amantadine for the CB[8] macrocycle was constructed.^237^ Indirect fluorescence detection of amantadine was based on a competitive reaction with the fluorescent probe for the inclusion within the CB[8] cavity, but was carried out in a flow injection spectrophotometric analysis system (FIA) (Fig. 29b). This method allows for detection of amantadine in pharmaceutical formulations and in human serum samples with excellent recoveries, ranging from 83 to 98%, depending on the nature of the matrix assayed. Even more, the selectivity of the method was evaluated against different antiviral drugs: rimantadine, acyclovir and ribavirine. According to the authors, this was the first time that a CB[*n*] supramolecular system has been employed for an analytical determination in a FIA system.

In 2019 Biedermann *et al.* published IDAs based on CB[8] that were capable of detecting memantine in a blood serum.^239^ The operational system was designed after mathematical simulations and a new class of [2.2]paracyclophane-derived indicator dyes was developed, possessing both excellent binding for the CB[8] host as well as good spectroscopic properties. Potential competitive binding of biofluids through the IDA was therefore overcome and quantification of memantine in blood serum could be successfully achieved. Afterwards other covalently bound CB[*n*]-indicators for amantadine quantification in biofluids were also designed, surpassing fundamental limitations of IDA like impractically slow equilibration time.^241,242^

**Fig. 30:**
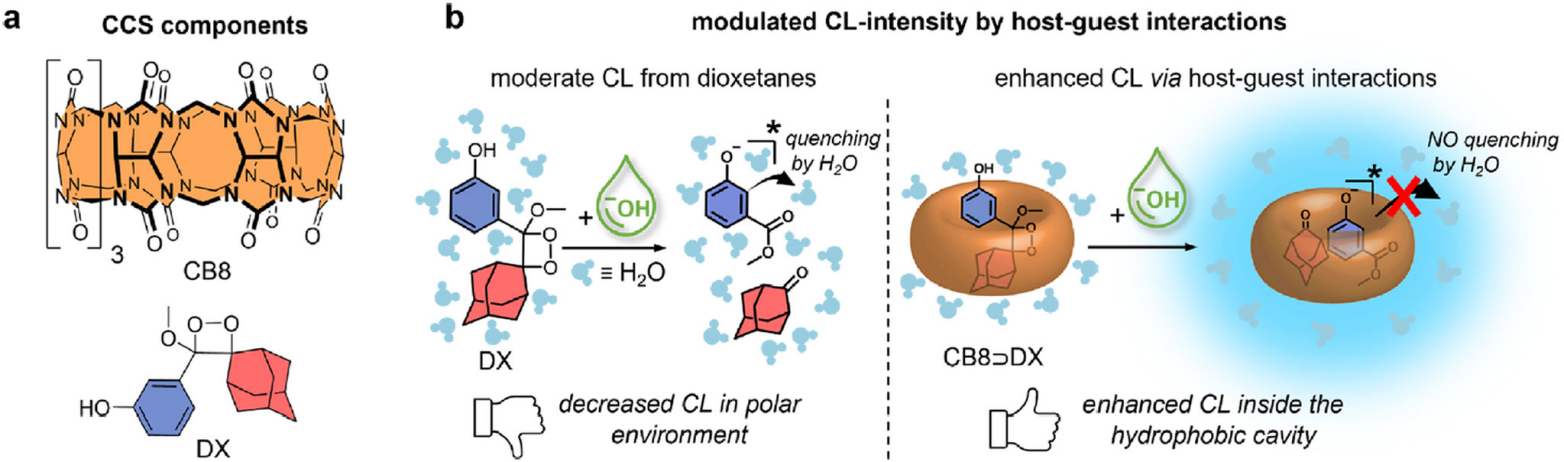
Illustration of the host and guest structures (a) and the role of their interactions in a supramolecular assay based on a chemiluminescent chemosensor (b) possessing an ADA subunit. Reproduced with permission from ref. 240, American Chemical Society, 2022.

Another illustrative example of two-fold implementation is an ADA/CB[*n*] system where an adamantyl-bearing 1,2-dioxetane acts as a chemosensor enclosed into the CB[*n*] cavity and also works as IDA for low micromolar memantine detection in human urine and human serum samples (Fig. 30).^240^ The authors showed that CB[8] can serve as a versatile chemiluminescent enhancer and moreover that this approach can be readily adaptable to a commercially available dioxetane, having the potential to improve chemiluminescence-based diagnostic assays.

Sensing of adamantane derivatives utilizing the ADA/CB[*n*] interactions was not achieved only through IDA assays^243^ but also by designing various sensors and by employing multidisciplinary analytical techniques and in continuation we will briefly mention a few related examples. A bioinspired smart nanochannel sensor based on competitive interactions for the sensitive and selective recognition of 1-aminoadamantane based on the ADA/CB[*n*] system was recently constructed.^244^ Specific host–guest interactions on the modified nanochannel led to the change of hydrophobicity and surface charge of the nanochannel that was observed as a change in ionic current, thus enabling high discrimination over other analytes and leading to sensitivity as high as 4.54 nM for 1-aminoadamantane recognition. Furthermore, a graphene and CB[7] electrode was developed for the differential pulse voltammetry (DPV) determination method of amantadine in biological fluids.^245^ Another example showcased that different CB[*n*] macrocycles with their specific portal-based host–guest interactions played a crucial role in promoting the formation of carbon dots (CDs) with a tunable particle size and tunable fluorescence properties.^246^ It was pointed out that these systems could be further employed as fluorescent biosensors for 1-aminoadamantane with a limit of detection up to a nM level (4.65 × 10^−9^ M) in a water environment.

We can conclude that implementation of the ADA/CB[*n*] system in combination with various analytical methods shows the advantages of using naturally occurring selective intermolecular interactions, which are happening on a molecular level, to obtain observable and measurable macroscopic responses that are of practical use.

### 3.6. ADA/CB[*n*] in sensing assays

**Fig. 31:**
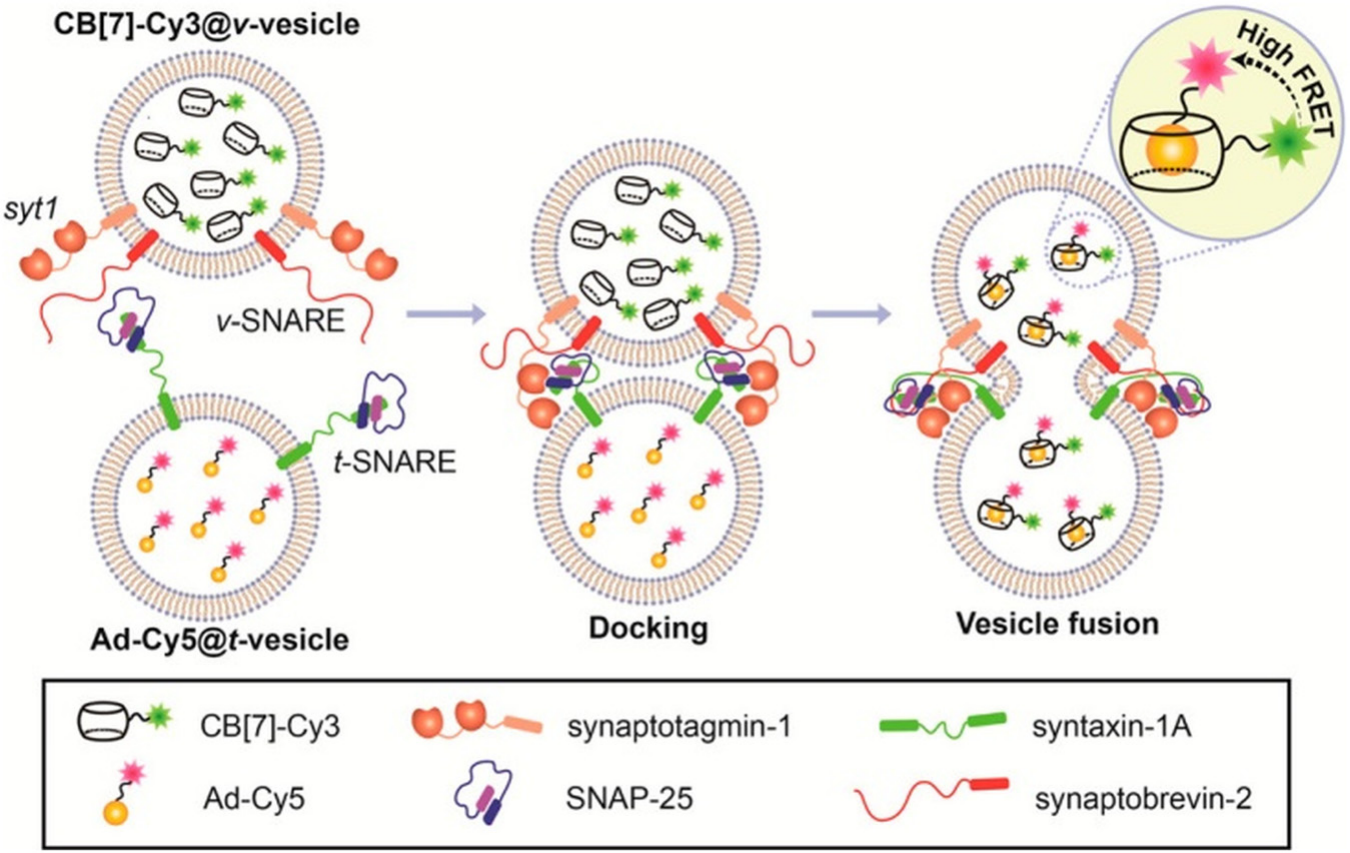
SNARE-mediated content mixing using a host–guest FRET pair: ν-vesicle containing Cy3-CB[7] and t-vesicle containing Ad-Cy5 undergo fusion and the resulting content mixing is detected by a FRET signal. Reproduced with permission from ref. 247, American Chemical Society, 2015.

Supramolecular systems are often employed in biosensing and diagnostic applications in medicinal and pharmaceutical fields. As we already described in the previous section, the ADA/CB[*n*] supramolecular system in particular has many desirable characteristics, like its reliable synthesis, chemical and thermodynamic stability, small size, compact packaging, *etc.* Furthermore, its binding affinity is exploited thoroughly and processes of association and dissociation can be effectively controlled. Especially important in terms of the ADA/CB[*n*] application in bioimaging and biosensing is its bio-orthogonality in binding to biomolecules. For example, Kim and coworkers designed an ultratight binding complex consisting of a modified CB[7] with a cyanine 3 donor dye (Cy3-CB[7]) and of 1-aminoadamantane conjugated with a cyanine 5 acceptor dye (Ad-Cy5) that exhibited fluorescence resonance energy transfer (FRET) upon complexation (Fig. 31).^247^ This complex type was used to study mechanisms of biological processes like SNARE (soluble *N*-ethylmaleimide-sensitive factor attachment protein receptor) mediated membrane fusion. More specifically, Cy3-CB[7] and Ad-Cy5 were encapsulated in ν- and t-vesicles, respectively, and when fusion occurred energy transfer from Cy3 to Cy5 could be observed due to the nearing of these fluorescent Cy3 and Cy5 moieties mediated through the encapsulation of the ADA moiety within the CB[7] cavity. With the help of fluorescent molecular probes, detection of pore opening and content release/mixing could be reliably done on a macroscopic level. The authors emphasized that this was the first successful sensing of flickering dynamics of a fusion pore by an *in vitro* assay using neuronal SNARE-reconstituted vesicles.^247^

**Fig. 32:**
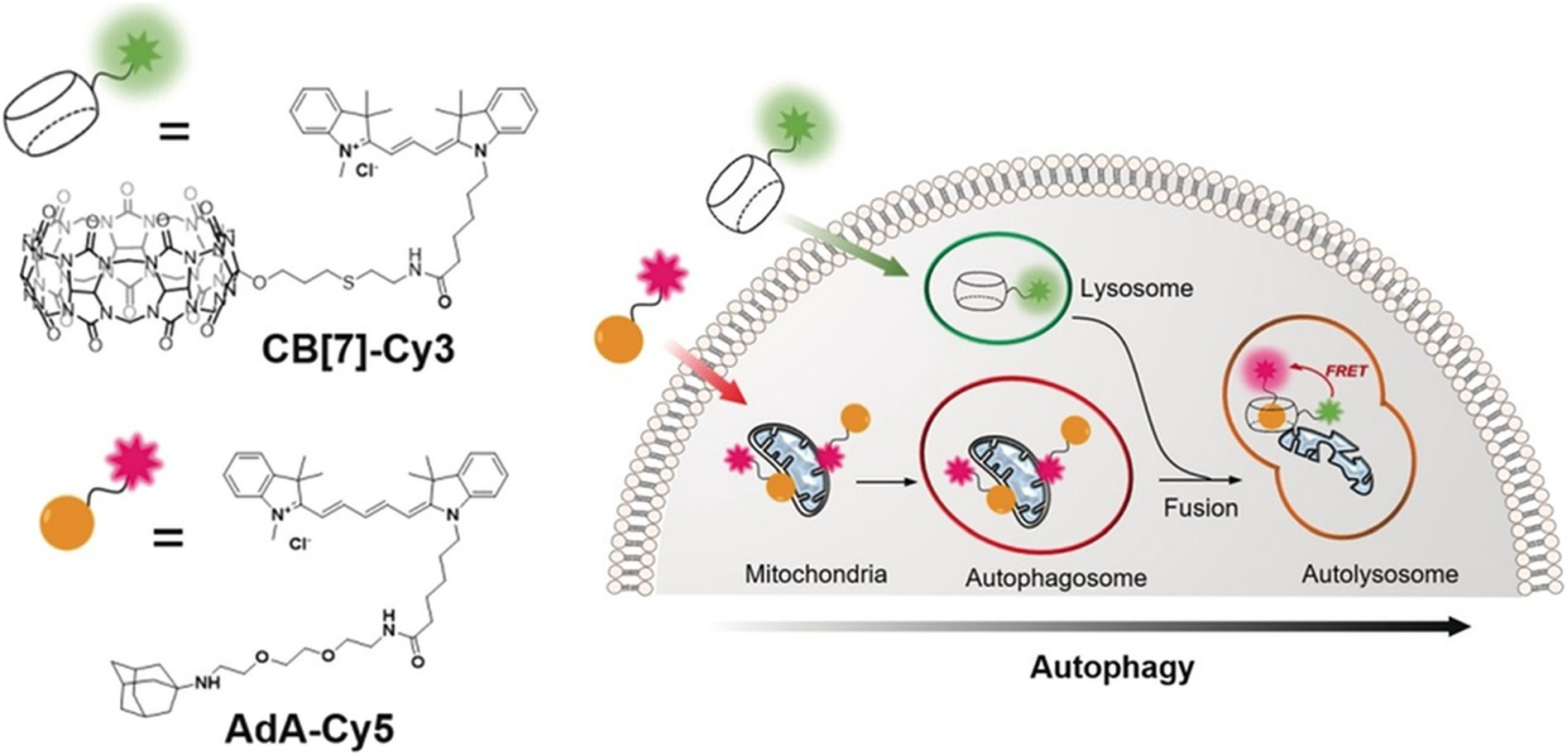
Emergence of FRET after the fusion of two different organelles (lysosome and mitochondria) through the process of autophagy. Reproduced with permission from ref. 248, Wiley VCH, 2018.

In the next example, the same molecular probe FRET pair was helpful in visualization of intracellular processes such as autophagy in living cells.^248^ By using confocal laser scanning microscopy it was observed that Cy3-CB[7] and Ad-Cy5 crossed the membrane and accumulated in lysosomes and mitochondria, respectively (Fig. 32). Upon complexation of the ADA moiety into the CB[7] cavity a FRET signal emerged as a result of the fusion of lysosomes and mitochondria. In that way not only can the FRET signal event be observed but the host–guest system can also be exploited as a new tool for imaging cellular processes in living cells.

Very recently the same principle was applied for a rational design of supramolecular FRET pair consisting of Cy3-CB[7] and boron-dipyrromethene 630/650-adamantylammonium (BDP-AdA), with a purpose to visualize lipophagy.^249^ The authors showed how intracellular accumulations of Cy3-CB[7] in lysosomes and BDP-AdA in lipid droplets resulted in FRET signals upon formation of an inclusion complex between Cy3-CB[7] and BDP-AdA, thus enabling visualization of the lipid droplets fusion with lysosomes (lipophagy). Again the ADA/CB[7] system was utilized but now with a different fluorescent dye conjugated to the lipophilic ADA moiety, which served to demonstrate a diversity of substrates that could be built into molecular probes for various live imaging purposes.

**Fig. 33:**
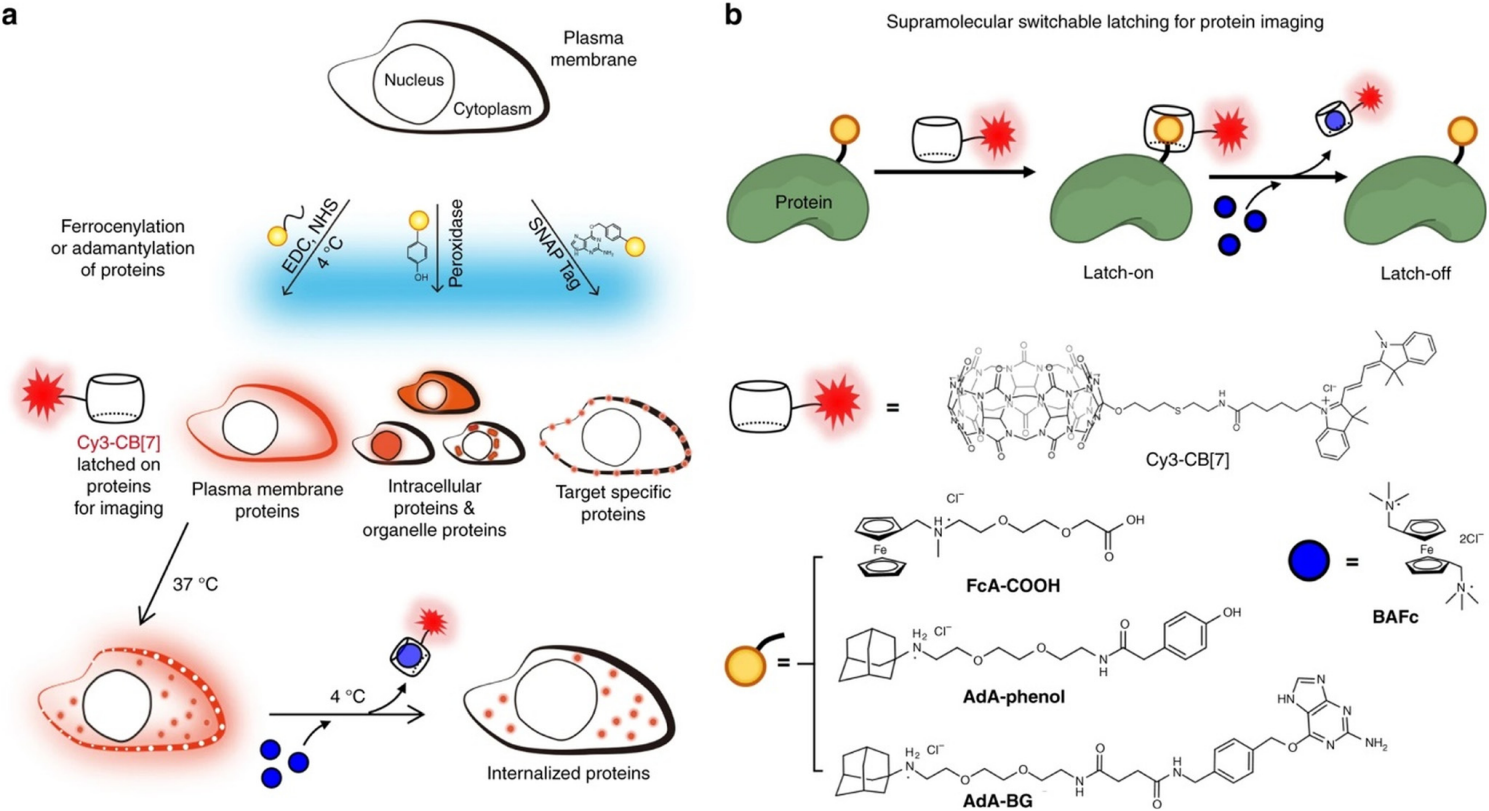
Universal and selective imaging of cellular proteins using a switchable supramolecular latching system (a) based on the interactions between Cy3-CB[7] and ADA derivatives (b). Reproduced with permission from ref. 250, Nature Research, 2018.

This same supramolecular approach was also used for fluorescence imaging of proteins located in and on fixed or living cells.^250^ The proteins of interest were first chemically or biologically conjugated with ferrocene or ADA derivatives and afterwards Cy3-CB[7] was latched to proteins *via* intermolecular interactions acting during the process of inclusion of 1-aminoadamantyl (AdA) or ferrocenemethyl (FcA) moieties into CB[7] (Fig. 33). By applying fluorescence microscopy it was possible to obtain clear fluorescence images needed for accurate and precise analysis of protein locations. Control of the system was elegantly achieved by treatment with a stronger guest competitor for the CB[7] cavity. At low temperature this allows for a selective release of Cy3-CB[7] from AdA or FcA labeled proteins on the cell surface. The fluorescent signal of proteins on cellular surfaces vanishes whereas that for the internalized proteins remains. The authors showcased the advantages of using such a synthetic system, *i.e.*, an ultrastable complex formed between Cy3-CB[7] and FcA or AdA conjugated proteins, when compared to the streptavidin-biotin (SA-BT) pair. The latter is a natural protein-based binding pair which is very sensitive in cellular environment and prone to enzymatic degradation.

**Fig. 34:**
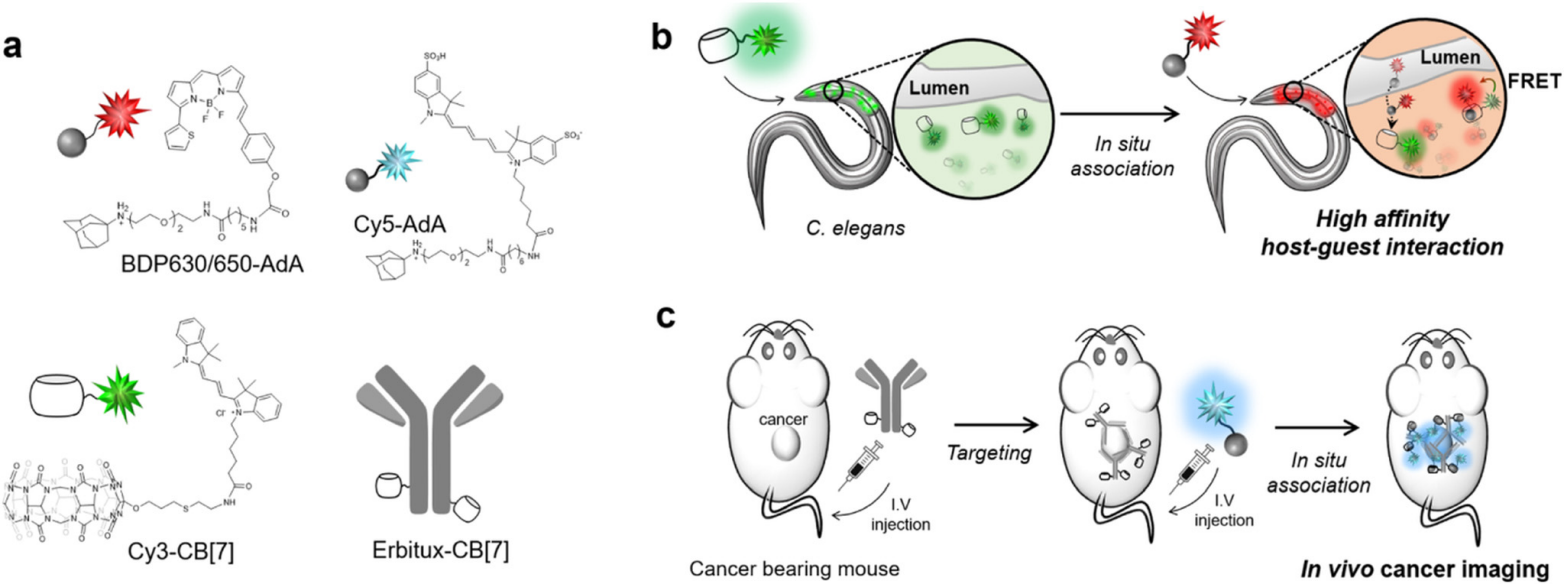
Locations of high-affinity host–guest interaction sites (a) in live *C. elegans* (b) and *in vivo* cancer imaging using the interaction between Erbitux-CB[7] and AdA-Cy5 that are sequentially injected to a cancer-bearing mouse through the tail vein (c). Reproduced with permission from ref. 251, American Chemical Society, 2019.

Kim's group even applied the same principle of non-covalent *in situ* anchoring of small synthetic molecules in a live organism (worm, *Caenorhabditis elegans*) by using a fully synthetic high-affinity binding pair consisting of Cy3-CB[7] and BODIPY BDP630/650-AdA (Fig. 34).^251^ What is more, it was also demonstrated that *in vivo* cancer imaging in a live mouse could be achieved using supramolecular latching of the synthetic system consisting of cyanine 5-AdA (Cy5-AdA) on a prelocalized CB[7]-conjugating antibody. This novel approach inspired many scientists to pursue synthetic host–guest pairs consisting of CB[*n*]s and ADA,^252–254^ eventually resulting in promising findings in the bioimaging field and providing new chemical tools for precise analysis of complicated mechanisms in biology.^255^

**Fig. 35:**
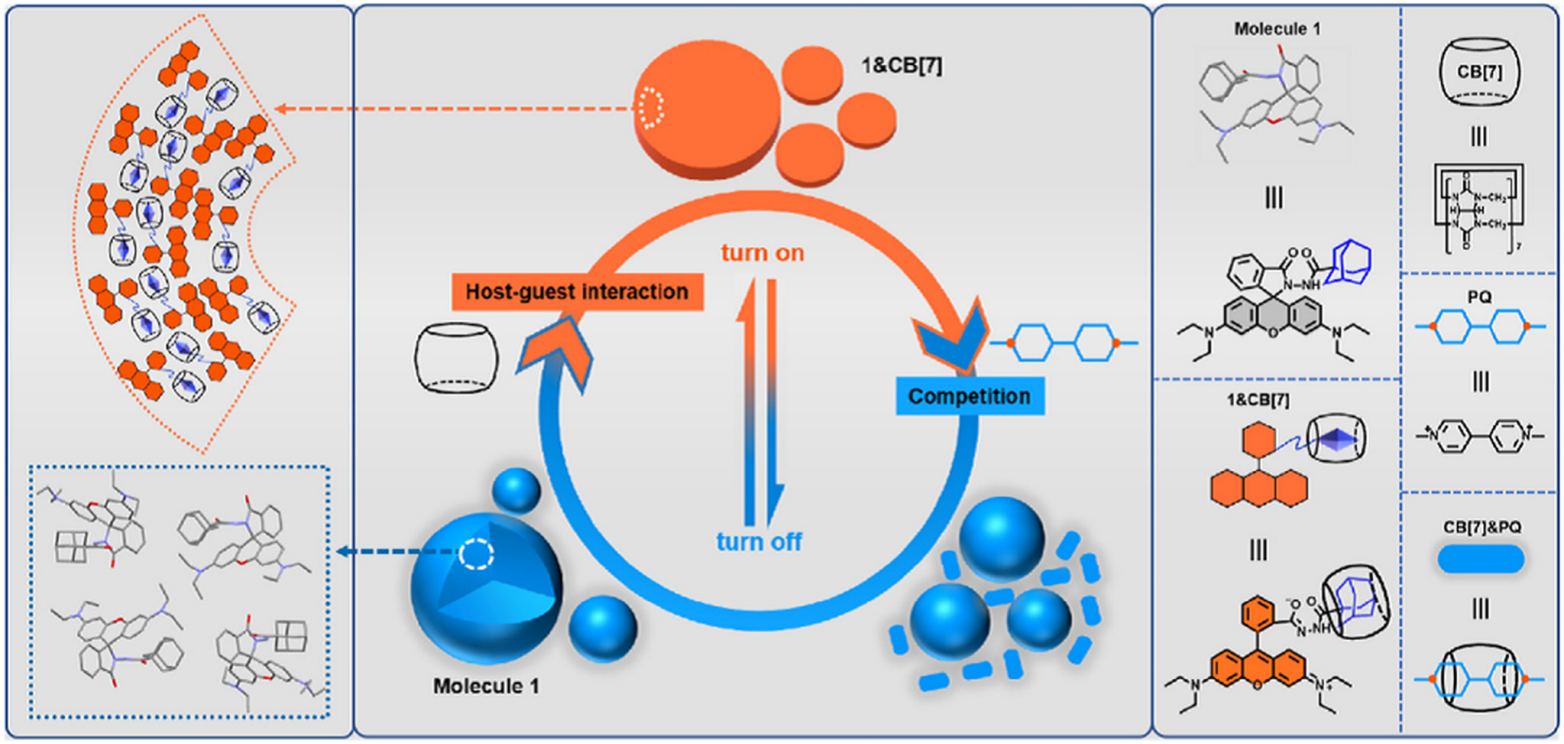
Illustration of complex formation and herbicide (paraquat, PQ) detection. Reproduced with permission from ref. 256, Elsevier, 2023.

As mentioned earlier, macrocyclic compounds have also found application as antidotes for toxins since upon encapsulation of those harmful agents their extreme toxicity towards non-target organisms is significantly reduced, especially towards animals and humans.^146,152,153^ An illustrative example to mention here is a highly fluorescent rhodamine-based probe for detection of herbicide paraquat (PQ) that was improved by utilizing the ADA/CB[7] system which had a stronger fluorescence intensity than rhodamine B itself.^256^ This feature could be attributed to the host–guest interaction which induced a spirocyclic ring-opening process and a suppression of host molecule aggregation. The complex in question displayed high sensitivity toward PQ, with an excellent nanomolar limit of detection (10 nM) and the authors pointed out that it could serve as a highly selective and sensitive probe for detecting PQ in various biological applications as well as in real life samples. It was concluded that this unique detection mechanism involved a process of self-assembly, disassembly and reassembly, which could be regenerated by the addition of host CB[7], thus providing a reversible fluorescence response to the analyte herbicide PQ (Fig. 35).

**Fig. 36:**
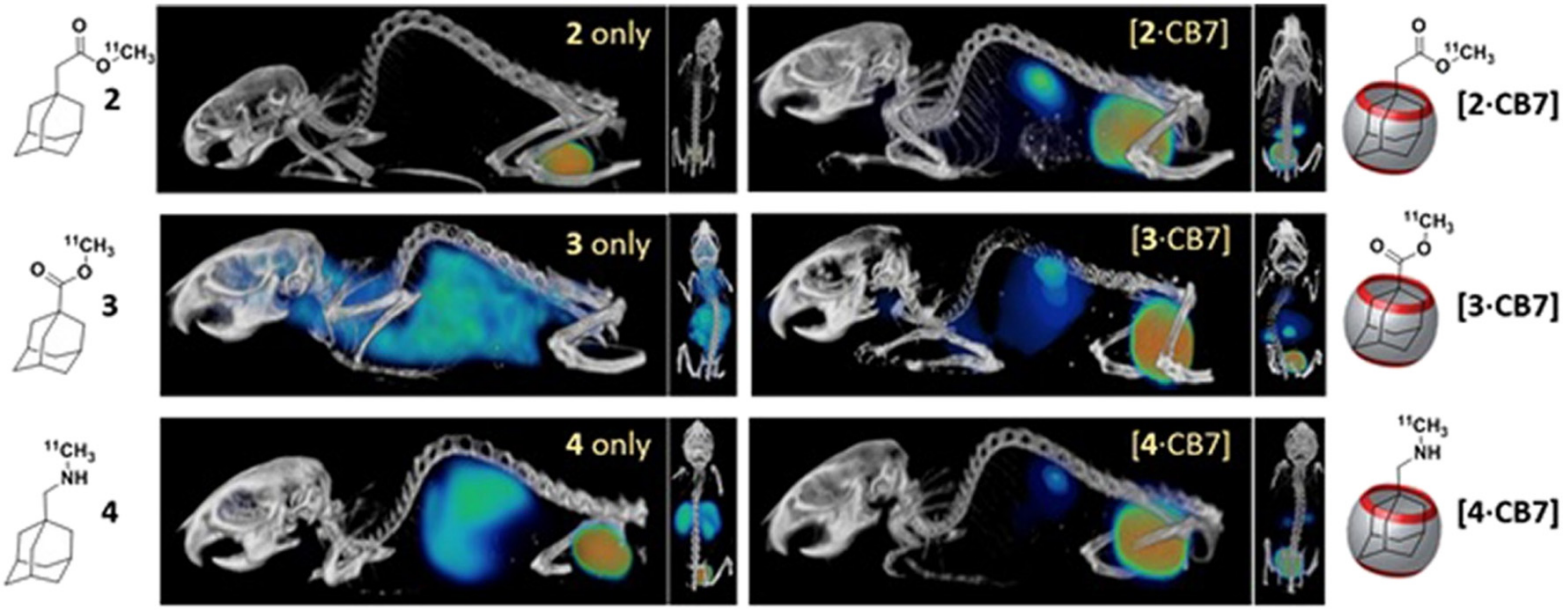
Immuno-PET imaging showing biodistribution of labeled ADA molecules in mice in the absence (left column) and presence (right column) of CB[7]. Reproduced with permission from ref. 257, Sage Publications, 2018.

On another note, in 2018 preliminary results dealing with the suitability of the ADA/CB[7] system in pre-targeted positron emission tomography (PET) imaging were presented (Fig. 36).^257^ The idea was that the CB[*n*]-conjugated antibodies could be visualized in the future by various radiolabeled ADA derivatives. This early proof-of-concept work shows that the ADA/CB[7] system itself (without antibody conjugation) is capable of remarkably fast complex formation and clearance *in vivo* and indeed has the potential for possible application in pre-targeted PET imaging procedures. This research direction was very recently further expanded on with the development of three ^64^Cu-labeled adamantane-containing guests/radioligands that were demonstrated to have high *in vitro* stability in combination with both specific and high tumor uptake.^258^

**Fig. 37:**
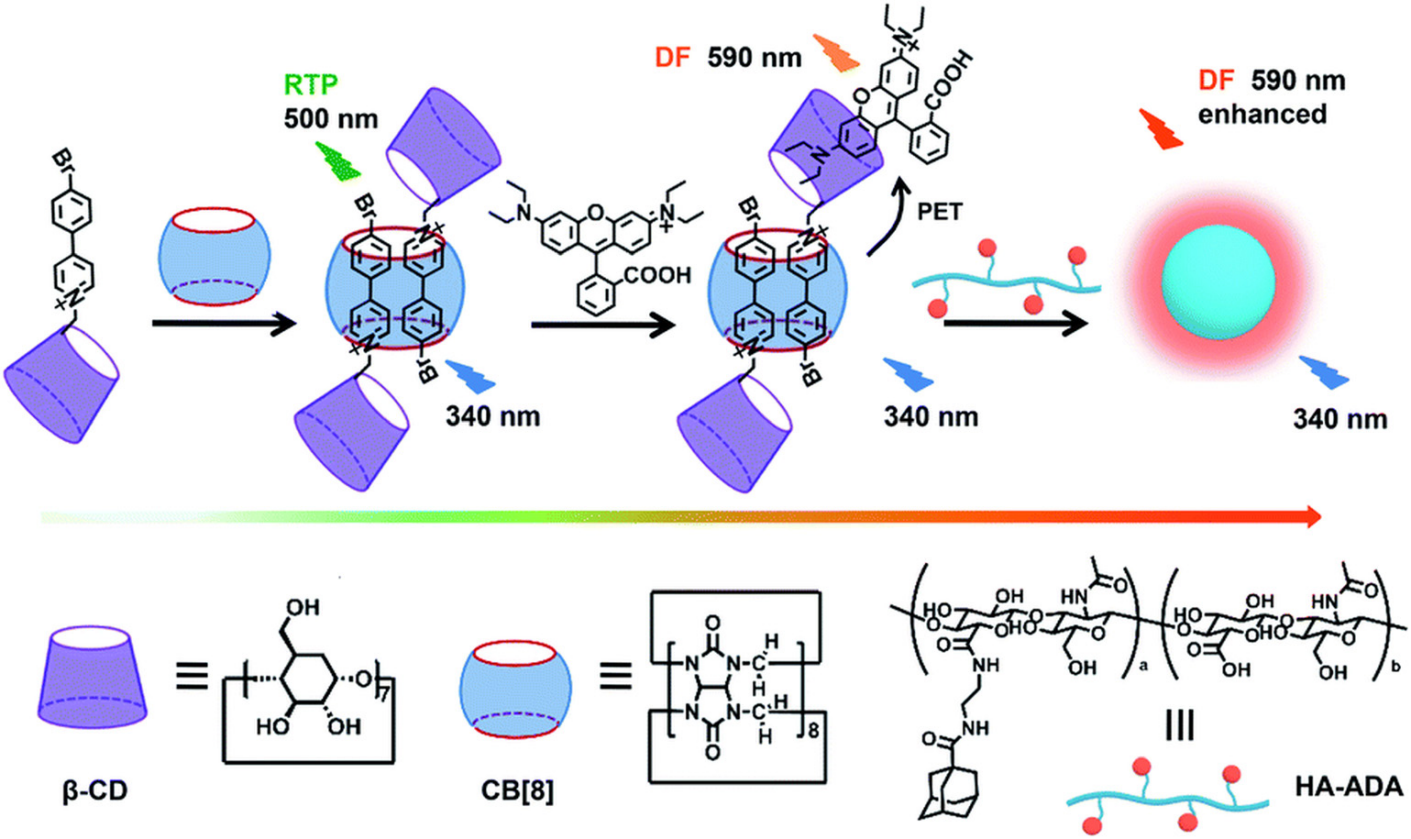
Construction of the supramolecular assembly for a purely organic light-harvesting PET system and related molecules. Reproduced with permission from ref. 259, the Royal Society of Chemistry, 2020.

A different example of the ADA/CB[*n*] use in bioimaging is a recently reported supramolecular assembly that was based on a purely organic light-harvesting phosphorescence energy transfer (PET) in an aqueous solution.^259^ It consisted of a pyridine modified β-cyclodextrin (CD-PY) as a donor, CB[8] as a mediator, rhodamine B (RhB) as an acceptor, and hyaluronic acid modified with the adamantane subunit (HA-ADA) as a cancer cell targeting agent (Fig. 37). In the first step pseudorotaxane is formed with CB[8] and CD-PY and the phosphorescence of CD-PY is “turned on” with a green phosphorescence emission at 510 nm. Afterwards, when RhB is encapsulated into the β-CD cavity, efficient solution-state light-harvesting PET occurs between pseudorotaxane and RhB, providing high energy transfer efficiency and an ultrahigh antenna effect between pseudorotaxane and RhB. However, the most important step is when HA-ADA assembles to form nanoparticles as it both promotes the delayed emission at 590 nm and successfully targets the mitochondria of A549 cancer cells.

### 3.7. Interdisciplinary applications and perspectives

Remarkably, in just 30 years since the exact structure of CB[*n*]s has been confirmed these macrocycles have been extensively studied in various systems of diverse biological applications. Simple host–guest systems, due to their promising features like bioavailability and non-toxicity, have the opportunity to dominate over other macrocycles in the fields of drug delivery and controlled drug release, with a possibility to further extend their sphere of influence to the fields of biosensing and diagnostic uses in medicine and pharmaceutical research. Additionally, CB[*n*] macrocycles in the role of host molecules^80^ could be implemented with other systems to build heterogeneous nano-assemblies, also with promising potential biological applications like drug and gene delivery, bioimaging, and photodynamic cancer therapy.^131,137,171,260–270^ In such hybrid systems CB[*n*]s are typically carrier vehicles for biologically active compounds or are functional parts of the emerging nanostructure and are therefore involved in controlling drug release mechanism from the nanomaterial. Up to now cucurbit[*n*]urils have successfully been combined with other nanomaterials^271^ like polymers,^272–276^ proteins,^131–133^ (*e.g.*, bovine serum albumin), metal nanoparticles,^277–281^*etc.* Even in these elaborate supramolecular assemblies the ADA/CB[*n*] system found its use to some extent, mostly for displacement purposes, and is applied either in compilation with heterogenic organic hybrids such as proteins^250^ or with organic–inorganic hybrids such as mesoporous silica nanoparticles (MSNPs),^282^ gold nanoparticles (AuNPs),^283,284^ zinc-doped iron-oxide nanoparticles,^285–287^*etc.* It should also be mentioned that inorganic nanoparticles usually have fascinating photo, thermal and magnetic properties or structural characteristics like porous morphology suitable for cargo loading, making them currently the leading research objectives in the field of drug delivery and bioimaging.

Host–guest interactions between functionalized ADA and CB[7] were also utilized in smart design of platelet-mimicking supramolecular nanomedicine (SN) for cascade amplification of synergistic chemotherapy. The applied supramolecular method proved to be superior because it allowed for exact control of the ratio of camptothecin (CPT) to cisplatin (Pt), two drugs which often could not be precisely regulated in different delivery systems.^288^ Among cucurbit[*n*]uril-based amphiphiles^289,290^ that self-assemble into nanomaterials as functional supramolecular micelles (FMS), many examples of ADA/CB[*n*] system could be employed for perspective biological and technological application,^291^ more specifically, for cell-targeted drug delivery^292^ or even as multifunctional theranostic agents for synergistic photodynamic therapy and hypoxia-activated chemotherapy.^293^ In these examples FSMs exhibited favorable characteristics such as high bioavailability, stability, precise targeting, and excellent delivery efficiency, making them highly promising for use in the drug delivery domain. In addition, high-affinity ADA/CB[*n*] interaction between functionalized DNA strands could direct hierarchical assembly of origami monomers into DNA nanofibers of micrometer-length.^294^

As another fascinating example of bioapplicable nanomaterial important for understanding and diagnosing various diseases, Wang and coworkers reported supramolecular luminol–AIEgen nanoparticles (SLA NP) used for bioimaging of deep-tissue-inflammation. The supramolecular system was constructed with ADA and CB[7] functionalized with luminol and AIEgen, respectively, using the resulting host–guest interactions to produce efficient bioluminescence resonance energy transfer (BRET) from blue-emissive luminol to red-emissive AIEgen, thus providing fluorescent imaging.^266^

**Fig. 38:**
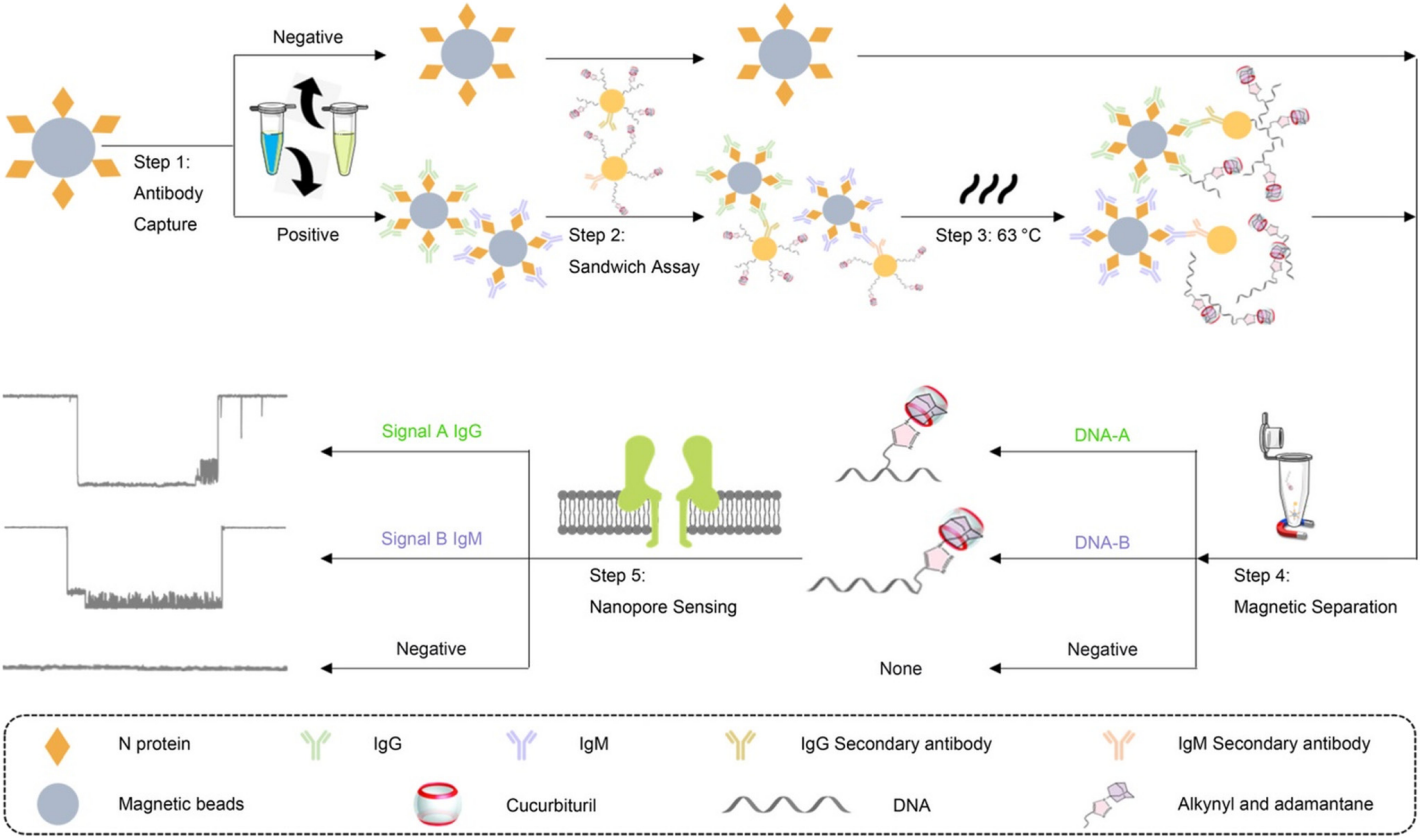
SARS-CoV-2 antibodies sensing: (1) capture of IgG and IgM antibodies with *N*-protein functionalized magnetic beads (MBs); (2) formation of the sandwich structure between IgG or IgM antibodies and probe DNA modified AuNPs; (3) thermal dehybridization of the probe DNAs from the AuNPs; (4) magnetic separation of probe DNAs from the sandwich structures; and (5) quantification of probe DNAs using the nanopore sensor. Reproduced with permission from ref. 295, Elsevier, 2021.

In the end, we will present one recent example of AuNPs combined with a different host–guest ADA/CB[*n*] supramolecular system that was developed for quantitative detection of SARS-CoV-2 antibodies in human serum.^295^ The sensing principle involves several steps (Fig. 37) and the highly specific response for SARS-CoV-2 antibodies is based on two characteristic signals obtained by translocations of ADA/CB[6]-modified probe DNAs through α-hemolysin (α-HL) (step 5 on Fig. 38). The signals in question differ from signals of other biomolecules and act as surrogates instead of directly measuring IgG or IgM antibodies. The authors claim that such DNA-assisted nanopore biosensor with two unique oscillating patterns in the signal can reliably quantify SARS-CoV-2 antibodies with high accuracy, large dynamic range, and potential for assay automation, despite the assay protocol consisting of multiple manual steps and long incubation times.^295^

## 4. Conclusion and outlook

Interest of medicinal chemists for the adamantane scaffold has recently expanded to the supramolecular ADA/CB[*n*] system. Such attention to adamantane derivatives as components of complex drug delivery systems stems from the fact that the adamantyl cage almost ideally occupies the corresponding CB[*n*] cavity, enabling this supramolecular system to be regarded by many as a golden standard in the field of host–guest mediated drug delivery. Through the selected recent examples we showcased how the ADA/CB[*n*] not only affects the bioactivity and bioavailability of drug molecules but can also tune drug properties. However, the applicability of this system goes beyond even that; implementation could be achieved also in the fields of practical bioapplication, bioanalytics, sensing assays, bioimaging, *etc.*

A good fit between the hydrophobic ADA and the CB[*n*] host cavity enables a strong and selective binding and affords stable inclusion complexes, which in turn opens up new venues for drug delivery. Namely, the emerging synergistic properties of such complexes in live cells give rise to exciting new pharmacological tools and offer an advantage over traditional single drug molecule approach. For example, host–guest non-covalent interactions are a useful and often affordable way to overcome existing physicochemical barriers hindering medicinal application of some drug candidates since supramolecular effects can facilitate the change of intrinsic properties of the drug (guest), like heightening its solubility, improving its chemical and thermal stability and metabolic resistivity by encapsulation, *etc.* The result is a focused delivery to a biological target and efficient drug action upon release, while at the same time reducing the amount of the active compound needed for a successful treatment. Non-covalent interactions responsible for guest encapsulation essentially enable active compound shielding and steric protection against metabolic processes like oxidation, hydrolysis and various nucleophilic attacks during the *in vivo* transport, thereby increasing the overall drug bioavailability. Furthermore, the host's macrocyclic cavity mimics the function of the binding region of a biological receptor with its pre-organized shape and by engaging in complexation with the guest the resulting ADA/CB[*n*] system becomes essentially a biomimetic entity. Here we should mention that one significant drawback to using CB[*n*] hosts as drug carriers or in other bioapplications that was identified is their somewhat poor water solubility. However, that could be successfully circumvented by chemical functionalization methods, by adding to the formulations biocompatible salts, or by changing the pH. Complexation induced shifting of the p*K*_a_ values of the guest molecules and their subsequent water solubility increase is especially useful for compounds that are readily protonated by nature. Additionally, the synergistic effect upon complexation is not limited solely to guest (drug) molecules; by inclusion complex formation the solubility of the supramolecular ADA/CB[*n*] system as a whole can often be increased to levels acceptable for practical purposes. Note that despite a higher binding preference of ADA towards CB[*n*] when compared to cyclodextrin hosts, drug release from the CB[*n*] cavity can still be effectively achieved by using thermodynamic means since complex formation is a dynamic process governed by non-covalent interactions. A method commonly used for drug release from reversible supramolecular systems is introduction of a second molecule with higher affinity for the host, aptly termed guest displacement. However, one future challenge of this field is to develop new ADA/CB[*n*] variations capable of achieving a good balance between stable encapsulation and efficient release at biological target sites. Supramolecular chemotherapy, encapsulation of inherently toxic bioactive compounds in order to decrease their cytotoxicity to healthy cells, is also a principle readily used for integrating non-covalent interactions in targeted drug therapy.

Applications of ADA/CB[*n*] in chemosensing in physiologically relevant biological systems have also been on the rise in recent years but there still remain many challenges to be overcome due to very low analyte concentrations as well as competitive substrate interferences. Bioanalytical and diagnostic applications are also of interest and significant headways have been made in these fields as well since the ADA/CB[*n*] system is capable of high selectivity and strong binding which then, with careful design, translates to specific interactions and bio-orthogonality in binding. For example, application of CB[*n*]-based molecular sensors as indicator displacement assays for ADA derivatives detection in a biological environment (*e.g.*, in urine, human serum, blood serum, biofluids, *etc.*) has gained significant attention since the technique can provide an accurate and specific observable response, unlike direct binding assays.

Future implementation of the ADA/CB[*n*] in the fields of drug delivery and controlled drug release depends heavily on the design of bioactive guest molecules with an ADA scaffold as a structural feature responsible for enabling the binding event. Intracellular recognition and subsequent action of the bioactive molecule is ultimately the purpose of the supramolecular system where the CB[*n*] host acts as a vehicle. In areas like biosensing and other diagnostic uses in medicine and pharmaceutical research the challenge remains to identify suitable *in vivo* targets and then develop the supramolecular complex which utilizes the ADA–CB[*n*] interaction and which is capable of providing a specific, accurate and reliable response.

Other exciting areas where ADA/CB[*n*] shows a potential for future bioapplication are in hybrid heterogeneous nano-assemblies for drug and gene delivery, bioimaging, photodynamic cancer therapy, *etc.* In such elaborate supramolecular assemblies the ADA/CB[*n*] may be used for displacements purposes, as a cargo vehicle and beyond. Taking into account all the current applications of the ADA/CB[*n*] system as well as the possibilities currently in development, one cannot but conclude that the future looks supramolecular!

## Author contributions

The manuscript was written through contributions of both authors. Both authors have given approval to the final version of the manuscript.

## Conflicts of interest

The authors declare no competing financial interest.

## Supplementary Material
